# Potentiating Therapeutic Effects of Epidermal Growth Factor Receptor Inhibition in Triple-Negative Breast Cancer

**DOI:** 10.3390/ph14060589

**Published:** 2021-06-18

**Authors:** Kyu Sic You, Yong Weon Yi, Jeonghee Cho, Jeong-Soo Park, Yeon-Sun Seong

**Affiliations:** 1 Department of Biochemistry, College of Medicine, Dankook University, Cheonan 31116, Chungcheongnam-do, Korea; kisuhezu@gmail.com; 2 Graduate School of Convergence Medical Science, Dankook University, Cheonan 3116, Chungcheongnam-do, Korea; 3 Department of Nanobiomedical Science, Dankook University, Cheonan 31116, Chungcheongnam-do, Korea; dragon101@gmail.com (Y.W.Y.); jeonghee.cho@dankook.ac.kr (J.C.)

**Keywords:** anticancer, combination therapy, epidermal growth factor receptor (EGFR), anti-EGFR therapy, EGFR inhibitor (EGFRi), EGFRi resistance, triple-negative breast cancer (TNBC)

## Abstract

Triple-negative breast cancer (TNBC) is a subset of breast cancer with aggressive characteristics and few therapeutic options. The lack of an appropriate therapeutic target is a challenging issue in treating TNBC. Although a high level expression of epidermal growth factor receptor (EGFR) has been associated with a poor prognosis among patients with TNBC, targeted anti-EGFR therapies have demonstrated limited efficacy for TNBC treatment in both clinical and preclinical settings. However, with the advantage of a number of clinically approved EGFR inhibitors (EGFRis), combination strategies have been explored as a promising approach to overcome the intrinsic resistance of TNBC to EGFRis. In this review, we analyzed the literature on the combination of EGFRis with other molecularly targeted therapeutics or conventional chemotherapeutics to understand the current knowledge and to provide potential therapeutic options for TNBC treatment.

## 1. Introduction

The epidermal growth factor receptor (EGFR) family (also known as the human epidermal growth factor receptor (HER) or erythroblastic leukemia viral oncogene homolog (ERBB) family) belongs to the receptor tyrosine kinase (RTK) superfamily, which consists of 58 transmembrane RTK proteins [1]. The EGFR/ERBB family members include EGFR (HER1/ERBB1), HER2 (ERBB2), HER3 (ERBB3), and HER4 (ERBB4) [2,3,4,5]. Under normal physiological conditions, the EGFR family members are activated by homo- and heterodimerization among them induced by their cognate ligands, such as epidermal growth factor (EGF), transforming growth factor alpha (TGF-α), or amphiregulin (AREG), binding to their ectodomain (extracellular domain) [2,6]. After ligand binding, the activated EGFR dimer triggers multiple downstream signaling pathways, including the phosphatidylinositol-3-kinase (PI3K)/v-akt oncogene homolog (AKT)/mammalian target of rapamycin complex 1 (mTOR1) pathway, the rat sarcoma (RAS)/v-raf-1 murine leukemia viral oncogene homolog (RAF)/MAPK/ERK kinase (MEK)/extracellular signal-regulated kinase (ERK) pathway, and the Janus kinase (JAK)/signal transducer and activator of transcription 3 (STAT3) pathway, which are known to play key roles in various cellular responses such as cell proliferation, cell cycle progression, cell survival, and cell motility [2,6,7,8,9]. Aberrant activation of EGFR caused by various alterations such as copy number amplification, mutations, and protein overexpression has been reported in the majority of human cancers, including bladder, breast, colon, head and neck, kidney, liver, lung, ovary, pancreas, prostate, and stomach cancers [10]. In addition, inhibition of the oncogenic activity of EGFR with targeted drugs has been proven to be an effective clinical strategy in treating tumors driven by abnormal EGFR activation. However, the efficacy of this clinical tactic is quite limited due to the rapid emergence of drug resistance in patients following drug treatment. In lung adenocarcinoma, several resistance mechanisms of EGFR-targeted drugs such as erlotinib and osimertinib (EGFR-targeted small-molecule inhibitors) have been well established [11,12]. These include acquisition of additional mutations to EGFR such as T790M and C797S as resistance mechanisms to erlotinib and osimertinib, respectively [13,14]. Several concurrent genomic alterations including *MET* amplification or anexelekto (AXL) overexpression have also been identified as major drug resistance mechanisms [15].

Triple-negative breast cancer (TNBC) is clinically defined as a breast cancer subtype that lacks expression of the estrogen receptor (ER) and progesterone receptor (PR) and has no amplification of HER2 [16,17]. TNBC accounts for approximately 15–20% of diagnosed breast cancers [16,17,18,19,20,21]. However, few targeted therapies with limited clinical outcomes have been approved for TNBC treatment [22]. In addition, more than 50% of cases of TNBC are known to express a high level of EGFR, which is associated with a poor prognosis [4,5,16,21,23,24]. EGFR expression has also been implicated with an unfavorable response to chemotherapy in patients with TNBC [25]. TNBC has been classified into at least six molecular subtypes, including basal-like 1 and 2 (BL1 and BL2), immunomodulatory (IM), luminal androgen receptor (LAR), mesenchymal (M), and mesenchymal stem-like (MSL) subtypes [19,26]. This classification was further refined into four subgroups, including BL1, BL2, M, and LAR, using histopathological quantification and laser-capture microdissection of clinical samples, since the IM and MSL subtypes have been identified to be contributed from infiltrating lymphocytes and tumor-associated stromal cells, respectively [27]. Continuous efforts to stratify molecular subtypes of heterogenous TNBC are still ongoing (reviewed in [28,29,30]).

Although activated EGFR signaling is observed in the BL2 and MSL subtypes of TNBC [19], TNBC has intrinsic resistance to anti-EGFR therapies [31], which has been supported by the disappointing outcomes of earlier attempts to treat TNBC with anti-EGFR monotherapies [32,33,34,35]. Thus, these results suggest that alternative oncogenic signaling initiated by receptors or downstream effectors may be the potential mechanism associated with the inefficacy of EGFR-targeted therapy against TNBC [36]. Consistent with this notion, various drug combination strategies to overcome resistance to EGFR-targeted drugs are currently under investigation.

In this report, we reviewed the recent progress of combination approaches related to anti-EGFR therapies for TNBC in 73 published studies. These publications were further analyzed to explore the current knowledge on the therapeutic windows of potentiating EGFR inhibition using drug combinations for TNBC treatment. Since multigeneration EGFR tyrosine kinase inhibitors (TKIs) and anti-EGFR antibody therapeutics have already been approved, the development of a combination strategy may provide an alternative therapeutic option to treat TNBC.

## 2. Anti-EGFR Therapeutics

**Figure 1:**
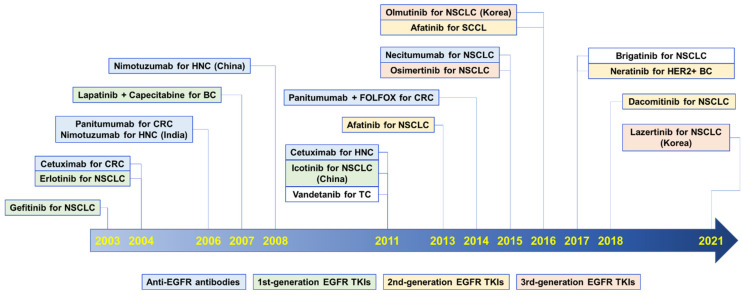
Milestones of anti-EGFR therapeutics approved globally. Some important milestones of regulatory approval for EGFR inhibitors are presented. See Table 1 and Table 2 for more details. If not specified in parentheses, anti-EGFR therapeutics were approved by the US Food and Drug Administration (US FDA). Abbreviations: BC, breast cancer; CRC, colorectal cancer; HNC, head and neck cancer; NSCLC, non-small cell lung cancer; TC, thyroid cancer; TKIs, tyrosine kinase inhibitors.

To date, four anti-EGFR antibodies and twelve EGFR TKIs have been approved globally for treatment of various human cancers (Figure 1).

### 2.1. Anti-EGFR Antibody Therapeutics

Currently, three anti-EGFR antibodies have been approved by the US Food and Drug Administration (FDA), including cetuximab (Erbitux^®^), panitumumab (Vectibix^®^), and necitumumab (Portrazza^®^) (Table 1) [37]. All of these antibody therapeutics are infused intravenously over the recommended time period [38,39]. Therapeutic anti-EGFR antibodies have been proposed to suppress the enzymatic activity of EGFR by the following mechanisms: (1) blockage of ligand binding to EGFR; (2) steric inhibition of homo- or heterodimerization among EGFR family members; (3) enhancement of EGFR internalization and subsequent degradation; (4) induction of the complement-dependent cytotoxicity (CDC) pathway; (5) induction of G_1_ cell cycle arrest; (6) inhibition of proangiogenic factor expression; (7) induction of apoptosis; (8) induction of antibody-dependent cellular cytotoxicity (ADCC) by natural killer (NK) cells or monocytes or macrophages; or (9) induction of DNA damage [37].

Cetuximab is the first FDA-approved anti-EGFR antibody therapeutic. It is an IgG1 chimeric (mouse–/human) monoclonal antibody that potentially inhibits EGF from binding to EGFR and disrupts EGFR dimerization, leading to inhibition of its cognate downstream signaling activation [40,41]. It also induces ADCC by NK cells and neutrophils [42].

Panitumumab is the first fully human IgG2κ monoclonal antibody specific to the extracellular ligand-binding domain of EGFR, blocking the downstream signaling pathways [43]. The threshold level of EGFR for antitumor effects of panitumumab was found to be more than 17,000 receptors per cell in xenograft models using human cancer cells [44]. Panitumumab is the only FDA-approved IgG2 anti-EGFR antibody, and unlike cetuximab, it does not induce ADCC [42].

Necitumumab is the most recently FDA-approved IgG1 monoclonal anti-EGFR antibody and is used in combination with gemcitabine and cisplatin for the first-line treatment of patients with advanced squamous NSCLC [39]. Necitumumab is an EGFR antagonist that binds to EGFR and inhibits the binding of EGFR to its ligands, preventing EGFR dimerization and activation. Necitumumab has also been demonstrated to induce ADCC in EGFR-positive cells in vitro [39].

Nimotuzumab is a humanized IgG1 monoclonal anti-EGFR antibody that has been approved for the following cancer types: (1) squamous cell carcinoma of the head and neck (SCCHN) in India, Cuba, Argentina, Colombia, Ivory Coast, Gabon, Ukraine, Peru, and Sri Lanka; (2) pediatric and adult glioma in Cuba, Argentina, Philippines, and Ukraine; (3) nasopharyngeal cancer in China [45]. Similar to other IgG1 anti-EGFR antibodies, it blocks ligand binding to EGFR, exerting an antitumor effect, and can also induce ADCC [42,45,46].

**Table 1:** Approved anti-EGFR antibody therapeutics.

First Appr	Anti-EGFR Antibodies	*K_D_*^1^ in nM	Developer	Indications Approved by the US FDA ^2^	
2004	Cetuximab (Erbitux^®^, ICM-C225)	0.201 [40]; 1.8 [46]	ImClone Systems	2004	Advanced CRC ^3^
				2011	Late-stage HNC ^4^
2006	Panitumumab (Vectibix^®^, ABX-EGF)	0.05 [47]	Abgenix	2006	Metastatic CRC
				2014	First-line use of panitumumab and FOLFOX for wild-type KRAS metastatic CRC
				2017	Wild-type KRAS metastatic CRC
2006	Nimotuzumab (TheraCIM, h-R3, BIOMAb EGFR)	21 [46]	CIMYM BioSciences	2006	HNC by the Central Drugs Standard Control Organization (CDSCO), India
				2008	HNC by the China Food and Drug Administration (CFDA) ^5^
2015	Necitumumab (Portrazza^®^)	6.1 [48]	Eli Lilly	2015	Advanced squamous NSCLC

^1^*K_D_*, dissociation constant; ^2^https://www.drugs.com/, accessed on 15 April 2021; ^3^ CRC, colorectal cancer; ^4^ HNC, head and neck cancer; ^5^ https://www.pharmacodia.com/, accessed on 15 April 2021.

### 2.2. Small-Molecule TKIs

As of December 23, 2020, 62 small-molecule protein kinase inhibitors (PKIs) have been approved by the US FDA [49]. Among them, small-molecule TKIs for EGFR, including gefitinib, erlotinib, afatinib, osimertinib, and dacomitinib (Table 2), have been initially approved to treat non-small cell lung cancer (NSCLC) [50]. The EGFR/HER2 dual inhibitors, lapatinib and neratinib, which have similar half-maximal inhibitory concentration (IC_50_) values for EGFR and HER2, have been approved for patients with HER2-positive breast cancer (Table 2). To date, first-to-fourth-generation EGFR TKIs have been developed [51]. Unlike anti-EGFR antibodies, all EGFR TKIs are orally bioavailable [52], making it easy for patients to take the medicine.

#### 2.2.1. First-Generation EGFR TKIs

The first-generation EGFR TKIs are oral, reversible inhibitors that block the binding of adenosine triphosphate (ATP) to the tyrosine kinase domain [50]. These include gefitinib, erlotinib, lapatinib, and icotinib [50,51].

Gefitinib (Iressa^®^) is the first US FDA-approved small-molecule TKI [50]. It received accelerated US FDA approval in 2003 as a monotherapy for patients with advanced NSCLC after failure of both platinum and docetaxel therapies [50]. However, gefitinib was voluntarily withdrawn in 2012 due to its limited efficacy in subsequent clinical trials [50,53]. More recently, gefitinib received US FDA approval for the first-line treatment of patients with metastatic NSCLC with *EGFR* exon 19 deletion (ex19del) or exon 21 substitution (L858R) mutations [50,53]. This new approval is because clinical trials have demonstrated the efficacy of gefitinib in patients carrying *EGFR* mutations [54,55,56,57].

Erlotinib (Tarceva^®^) was discovered in 1997 as a selective EGFR inhibitor [58]. It was approved by the US FDA in 2004 to treat patients with advanced or metastatic NSCLC after the failure of at least one prior chemotherapy [59,60]. Due to its superiority compared with other chemotherapeutics in response and progression-free survival (PFS), erlotinib was further approved for the first-line therapy of patients with NSCLC with *EGFR* mutations [50].

Lapatinib (Tykerb^®^) is a potent dual EGFR/HER2 inhibitor [61]. Lapatinib was approved by the US FDA in 2007 for breast cancer in combination with capecitabine (Xeloda) [62]. Crystal structure analysis revealed that the conformation of lapatinib-bound EGFR is different from that of erlotinib-bound EGFR, leading to much slower dissociation of lapatinib from the complex than erlotinib [63]. Consistently, lapatinib downregulates tyrosine phosphorylation for much longer periods than erlotinib and gefitinib.

Icotinib (Conmana^®^) is another first-generation EGFR TKI that is solely approved (in 2011) and marketed in China for the second- or third-line therapy of metastatic NSCLC [50]. Icotinib is structurally similar to gefitinib and erlotinib but is less toxic and better tolerated than gefitinib [64]. In 2014, it was further approved for the first-line treatment of patients with *EGFR*-mutant NSCLC in China [65].

#### 2.2.2. Second-Generation EGFR TKIs

Second-generation EGFR TKIs are irreversible kinase inhibitors that covalently bind to the kinase domain of EGFR [66]. Afatinib (Tovok^®^, BIBW2992) is an ATP-competitive irreversible TKI that covalently binds and irreversibly blocks enzymatically active EGFR family members (EGFR, HER2, and HER4) [67]. It was approved by the US FDA in 2013 for late stage NSCLC, in 2016 for squamous cell carcinoma of the lung, and in 2018 for *EGFR* mutation-positive NSCLC (Table 2). The superiority of afatinib in terms of PFS and responses over pemetrexed plus cisplatin [68] or gemcitabine plus cisplatin [69] for patients with *EGFR*-mutant NSCLC was demonstrated in two clinical trials.

Neratinib (Nerlynx^®^) is an irreversible HER2 and EGFR TKI approved for the treatment of HER2-positive breast cancer [70]. In the EU and the US, neratinib was approved as an adjuvant treatment for adults with early-stage, hormone-receptor-positive, HER2-overexpressed or amplified breast cancer and adults with less than one year from prior adjuvant trastuzumab-based therapy [71]. In the US, neratinib in combination with capecitabine was also approved for adults with advanced or metastatic HER2-positive breast cancer [72].

Dacomitinib (Vizimpro^®^) is an irreversible pan-HER inhibitor (Table 2). It covalently binds to a cysteine residue in the ATP pocket of the kinase domain. Similar to afatinib, it blocks signal transduction from both homo- and heterodimers of all members of the EGFR/HER family [73,74]. Dacomitinib demonstrates longer pharmacodynamic effects than the first-generation TKIs [75].

#### 2.2.3. Third-Generation EGFR TKIs

To overcome the limited efficacy of second-generation EGFR TKIs for EGFR-activating mutations such as T790M, L858R, or ex19del, third-generation mutation-selective EGFR TKIs including alflutinib (AST2818), almonertinib (HS-10296), avitinib (AC0010), lazertinib (Leclaza^®^; YH25448), olmutinib (Olita^TM^), osimeritinib (Tagrisso^®^, AZD9291), narzatinib (EGF816), rociletinib (CO-1686), and WZ4002 have been developed [51,76].

Osimertinib is the first FDA-approved third-generation EGFR TKI for *EGFR*^T790M^ mutation-positive NSCLC and was approved in 2015 [76]. The T790M mutation may confer intrinsic (de novo or primary) or acquired (or secondary) resistance following first-line treatment with other EGFR TKIs, such as gefitinib and afatinib [13]. Although it is effective in treating locally advanced or metastatic NSCLC with T790M or other activating EGFR mutations [77], resistance to osimertinib usually occurs due to a C797S mutation approximately 10 months after treatment [14].

Olmutinib was approved in Korea in May 2016 for patients previously treated with an EGFR TKI with locally advanced or metastatic NSCLC with an *EGFR*^T790M^ mutation [78]. Olmutinib has been granted accelerated approval by the Korea Ministry of Food and Drug Safety (MFDS) under the condition that a phase 3 clinical trial would be conducted post-marketing. The US FDA also granted olmutinib a breakthrough therapy designation for NSCLC in December 2015 [78]. The C797S mutation of EGFR also develops after treatment with olmutinib [14].

Lazertinib was also conditionally approved in Korea in January 2021 for patients with NSCLC with *EGFR*^T790M^ mutations (Table 2). Lazertinib has been reported to have a higher selectivity and fewer off-target effects than osimertinib [79]. The phase 1–2 study of 127 patients in 14 hospitals in Korea reported a tolerable safety profile with a median PFS of 9.5 months for patients with *EGFR*^T790M^-positive NSCLC versus 5.4 months for those with *EGFR*^T790M^-negative NSCLC [80]. Resistance to lazertinib caused by the C797S mutation of EGFR has also been reported [81].

#### 2.2.4. Fourth-Generation EGFR TKIs

Allosteric inhibitors are under development as fourth-generation EGFR TKIs to overcome *EGFR*^L858R^, *EGFR*^T790M^, and *EGFR*^C797S^-mediated resistance [51]. BBT-176 is a first-in-class fourth-generation EGFR TKI designed to inhibit EGFR^C797S^ mutation that arises following osimertinib treatment in patients with NSCLC and is in a phase 1–2 clinical trial for advanced NSCLC [82]. The clinical trial was designed to use BBT-176 alone or in combination with cetuximab (NCT04820023). In preclinical studies, it showed strong antitumor activity in xenografts with *EGFR*^ex19del/T790M/C797S^ and *EGFR*^L858R/T790M/C797S^, and its efficacy was enhanced in combination with anti-EGFR antibody. BBT-176 was discovered by the Korea Research Institute of Chemical Technology (KRICT) and has been licensed by Bridge Biotherapeutics, with worldwide exclusive rights for further development.

EAI001 and EAI045 are non-ATP competitive inhibitors that bind allosterically to EGFR with higher specificity for mutant EGFR^T790M/C797S^ than for wild-type EGFR [83,84]. Another mutant-selective EGFR allosteric inhibitor, JBJ-04-125-02, blocks the EGFR^L858R/T790M/C797S^ signaling both in vitro and in vivo and has antitumor activity against an NSCLC xenograft tumor [85]. Further clinical benefits may be addressed in the future.

#### 2.2.5. Multitargeting TKIs

Examples of multitargeting TKIs with inhibitory activity against EGFR include brigatinib and vandetanib. Brigatinib (Alunbrig^®^; AP26113) is a multi-kinase inhibitor that was originally developed as a TKI against anaplastic lymphoma kinase (ALK) [86]. Brigatinib further inhibits multiple tyrosine kinases including proto-oncogene tyrosine protein kinase ROS, fms-related receptor tyrosine kinase 3 (FLT3), and insulin-like growth factor 1 receptor (IGF1R), with IC_50_ values of 1.9 nM, 2.1 nM, and 24.9 nM, respectively [86]. A drug screening against cells harboring *EGFR*^C797S/T790M/ex19del^ identified that brigatinib also has inhibitory activity against mutant EGFR including C797S/ex19del (IC_50_ = 39.9 nM), ex19del (IC_50_ = 43.7), C797S/T790M/ex19del (IC_50_ = 67.2), and T790M/ex19del (IC_50_ = 150.3) [87]. Brigatinib received accelerated approval by the US FDA in 2017 for ALK-positive (ALK+) metastatic NSCLC and was further approved in 2020 as a first-line treatment option for patients with ALK+ metastatic NSCLC (Table 2).

Vandetanib (Caprelsa^®^; Zactima^®^; ZD6474) is an orally available RTK inhibitor targeting vascular endothelial growth factor receptor 2/3 (VEGFR2/3), rearranged during transfection (RET), and EGFR, which are important targets in thyroid cancer (Table 2) [88,89]. It was the first FDA-approved medication for the treatment of advanced medullary thyroid cancer (MTC) [90]. Importantly, its in vivo antitumor effects are indirectly mediated by reducing the production of EGFR-induced angiogenic growth factor [89]. Vandetanib can prolong the PFS of patients with aggressive MTC and can stabilize the disease [90]. Vandetanib has been found to be effective in inducing in vivo tumor regression in TNBC PDX models with high expression of RET or EGFR with concordant suppression of RET/EGFR phosphorylation and MEK/ERK pathway activation [91]. Overexpression of RET was found in < 10% and 20–40% of TNBC- and HER2-positive breast cancer samples, respectively.

**Table 2:** Approved anti-EGFR TKIs.

First Appr	Gen ^1^	EGFRi	Known Targets (IC_50_ or EC_50_ nM)	Developer	Indications Approved by the US FDA ^2^	
2003	1st	Gefitinib (Iressa^®^, ZD-1839)	EGFR (37) [92]	AstraZeneca	2003	Advanced NSCLC ^3^
					2015	First-line treatment of metastatic NSCLC
2004	1st	Erlotinib (Tarceva^®^, OSI-774, CP358774, NSC 718781)	EGFR (2) [58]	OSI Pharmaceuticals	2004	NSCLC
					2010	Advanced NSCLC
					2013	First-line therapy of EGFR-mutant NSCLC [50]
2007	1st	Lapatinib (Tykerb^®^, GSK572016,GW2016)	HER2 (9.2), EGFR (10.8), HER4 (367) [61]	GlaxoSmithKline	2007	Advanced or metastatic breast cancer in combination with Xeloda (capecitabine)
					2010	First-line combination treatment of metastatic breast cancer
2011	-	Vandetanib (Caprelsa^®^, Zactima^®^, ZD6474, ZD6)	VEGFR2 (40), VEGFR3 (110), RET (130), EGFR (500) [88,89]	AstraZeneca	2011	Advanced medullary thyroid cancer
2011	1st	Icotinib (Conmana, BPI-2009H)	EGFR (5) [93]	Beta Pharma	2011	Solely approved for NSCLC by CFDA [50]
					2014	First line treatment for advanced NSCLC patients with EGFR mutation by CFDA [65]
2013	2nd	Afatinib (Gilotriff^®^, BIBW2992)	EGFR^L858^R (0.4), EGFR (0.5), HER4 (1), EGFR^L858R/T790M^ (10), HER2 (14) [94,95]	Boehringer Ingelheim	2013	late stage NSCLC
					2016	squamous cell carcinoma of the lung
					2018	EGFR mutation-positive NSCLC
2015	3rd	Osimertinib (Tagrisso^®^, AZD9291)	EGFR^ex19del/T790M^ (3), EGFR^L858R^ (9), EGFR^ex19del^ (12), EGFR^L858R/T790M^ (13) [96]	AstraZeneca	2015	EGFRT790M mutation-positive NSCLC
					2017	Full approval
					2018	First-line treatment for EGFR-mutated NSCLC
					2020	Adjuvant treatment of patients with early-stage, EGFR-mutated NSCLC
2016	3rd	Olmutinib (Olita^TM^, HM61713, BI 1482694)	EGFR^L858R/T790M^ (18) [78]; BTK (13.9), EGFR (17.6) [97]	Hanmi Pharmaceutical	2016	Approved for locally advanced or metastatic *EGFR*^T790M^-mutated NSCLC by the Korea MFDS ^4^ [78]
2017	-	Brigatinib (Alunbrig^®^, AP26113)	ALK (0.37), ROS1 (1.9), FLT3 (2.1) IGF1R (24.9), EGFR^C797S/ex9del^ (39.9), EGFR^ex19del^ (43.7), EGFR^C797S/T790M/ex19del^ (67.2) [86,87]	ARIAD Pharmaceuticals	2017	Accelerated approval for patients with ALK+ NSCLC
					2020	First-line treatment option for patients with ALK+ metastatic NSCLC
2017	2nd	Neratinib (Nerlynx^®^, HKI-272)	HER2 (59), EGFR (92) [70]	Puma Biotechnology	2017	Extended adjuvant treatment of early stage HER2+ breast cancer
					2020	HER2+ metastatic breast cancer
2018	2nd	Dacomitinib (Vizimpro^®^, PF00299804)	EGFR (6.0), HER2 (45.7), HER4 (73.7) [74]	Pfizer	2018	First-line treatment of *EGFR*-mutated metastatic NSCLC
2021	3rd	Lazertinib (Leclaza^®^, YH25448,GNS-1480)	EGFR^ex19del/T790M^ (1.7), EGFR^L858R/T790M^ (2), EGFR^ex19del^ (5.3), EGFR^L858R^ (20.6), EGFR (76) [79]	Genosco/Yuhan	2021	Approved for *EGFR*^T790M^-mutated NSCLC by the Korea MFDS

^1^ Gen, generation; ^2^ https://www.drugs.com/, accessed on 15 April 2021; ^3^ NSCLC, non-small cell lung cancer; ^4^ Ministry of Food and Drug Safety.

## 3. Resistance to Anti-EGFR Therapeutics

Resistance of cancer to anticancer therapeutics is classified into two main categories: intrinsic (de novo or primary) resistance and acquired (secondary) resistance [98]. Current knowledge and management strategies have recently been described in detail [98]. Here, we provide a brief overview of cancer resistance to anti-EGFR therapies, especially in TNBC.

TNBC has been demonstrated to have an intrinsic resistance to anti-EGFR therapies, including both anti-EGFR antibodies and EGFR TKIs [31]. For example, a study with 20 TNBC cell lines reported that twelve (60%) were classified as refractory to most of the 24 EGFR TKIs tested [99]. In addition, acquired resistance to anti-EGFR monotherapy has been reported in various types of cancers. For example, acquired resistance of NSCLC to EGFR inhibitors (EGFRis) develops within 9 to 14 months of treatment [85]. From the studies on NSCLC in which mechanisms of resistance to EGFRis have been extensively studied, intrinsic EGFRi resistance of TNBC may be due to (1) EGFR-activating mutations or amplification, (2) activation of bypass signaling pathways (e.g., *MET* amplification), or (3) activation of downstream pathways [50,76,77,100,101]. Rewiring of signal transduction pathways by dynamic signaling networks, including feedback loops and crosstalk, also contributes to unexpected adverse effects or EGFRi resistance in cancer cells [102,103].

### 3.1. EGFR Mutations or Amplification

Mutations conferring EGFR TKI resistance were discovered several months after EGFR TKI therapy [50,76,85,100,101]. T790M, the gateway mutation, was found in 50–65% of patients who had acquired resistance to EGFR TKIs [104,105,106]. This mutation blocks binding of first-generation TKIs to EGFR by increasing the binding affinity of EGFR to ATP [106]. In addition, a tertiary mutation, *EGFR*^C797S^, was detected in 20–25% of patients with NSCLC who received osimertinib [107,108]. C797 is the covalent binding site for all known second-generation EGFRis, while the C797S point mutation blocks the covalent binding of irreversible EGFR TKIs [107]. *EGFR*^C797S^-mediated resistance has also been reported in patients treated with olmutinib [109], rociletinib [110], and nazartinib [111].

In contrast to NSCLC, the incidence rate of *EGFR*-activating mutations in TNBC has been reported to be relatively low, at between 0% and 11.4% [112]. For example, a mutational analysis identified *EGFR* mutations in only 8 of 70 TNBC samples, with four samples demonstrating ex19 deletions, including 15-nucleotide deletions (del E746-A750) in 2 samples and 24-nucleotide deletions (del S752-I759) within the kinase domain in other samples. Furthermore, one sample showed inversion of the complementary strand of exon 19 and three samples showed exon 21 mutations, including one case of L858R and two cases of T847I [113]. Although positive staining for EGFR does not correlate well with *EGFR* mutations in these TNBC samples, the ex19del and exon 21 substitutions are commonly found in NSCLC and are good predictors of EGFR TKI sensitivity [114]. Additionally, no activating *EGFR* mutations were identified by tissue microarray and pyrosequencing analysis in 493 TNBC cases [112]. In fact, *EGFR* mutations are very rare in patients with TNBC in various populations. including American [115], Australian [116], British [117], French [118,119], German [120], Korean [112,121,122], Chinese [113,123], Switzerland [124], and Japanese [117,125,126] populations. Currently, intrinsic resistance of TNBC to anti-EGFR monotherapies has been understood to be a consequence of activation of a bypass or downstream signaling pathway(s) rather than due to *EGFR* mutations.

Accumulation of nuclear EGFR has been linked to drug resistance in TNBC [127,128,129,130]. Nuclear EGFR activates the transcription of genes involved in cell cycle progression, such as cyclin D1 [131] and aurora kinase A (*AURKA*) [132]. Nuclear EGFR, independent of canonical signaling pathway, has been reported to increase DNA repair in response to cisplatin and ionizing radiation by increasing nuclear accumulation and activation of the DNA-dependent protein kinase catalytic subunit (DNA-PKcs) [133]. DNA-PKcs plays a critical role in non-homologous end-joining DNA repair [134]. Interestingly, cetuximab and gefitinib have been reported to inhibit nuclear accumulation of EGFR, leading to cytoplasmic retention and inhibition of DNA-PKcs [135,136]. In addition, EGFR has also been associated with two essential components of the homology-directed recombinational repair (HDR), the RAD51 (*S. cerevisiae*) homolog (RAD51), and the breast cancer susceptibility gene 1 (BRCA1). The EGFRi gefitinib decreased the protein stability of RAD51 and its mRNA levels [137]. Erlotinib has also been demonstrated to decrease HDR by reducing RAD51 foci and increasing cytoplasmic BRCA1 [138].

### 3.2. Activation of Bypass Signaling Pathways

Although anti-RTK antibodies block the binding of the cognate ligands to the ectodomain of RTKs, leading to shutdown of downstream signaling pathways, agonist-like or partial agonist effects of RTKs have also been reported [139,140,141,142]. This partial agonistic action may induce autophosphorylation of RTKs that activate downstream signaling pathways, eventually causing resistance to anti-RTK antibodies. For example, trastuzumab and cetuximab exert agonistic effects on HER2 and EGFR, respectively [143,144,145]. In addition, the agonistic action of an anti-RTK antibody may transactivate other RKTs, such as IGF1R or VEGFRs [142].

Amplification or activation of other RTKs, such as mesenchymal epithelial transition factor (MET, also known as hepatocyte growth factor receptor (HGFR)), HER2, IGF1R, fibroblast growth factor receptor (FGFR), and AXL, might be a potential mechanism of intrinsic resistance to anti-EGFR monotherapies in TNBC [15]. Amplification of these RTKs drives activation of their downstream targets, such as the PI3K/AKT/mTORC1, RAS/RAF/MEK/ERK, and JAK/STAT3 pathways [98,146,147,148,149].

MET overexpression has been found in various cancers in association with a poor prognosis and confers resistance not only to therapies targeting EGFR, BRAF, and MEK, but also to chemotherapies [148]. High expression of MET in clinical TNBC samples has been associated with poor overall survival (OS) and disease-free survival (DFS) [150,151]. A previous study demonstrated that HGF, the ligand for MET, is secreted from fibroblasts and activates MET in TNBC cells, leading to EGFRi resistance through EGFR–MET crosstalk [152]. The fact that knockdown of *EGFR* in TNBC cells abolished the HGF-dependent TNBC cell survival further supports the importance of EGFR–MET crosstalk in TNBC cells [152]. Activation of MET by HGF has also been reported to promote resistance to EGFRis in TNBC cells [153].

HER2 has also been identified to induce drug resistance via activation of nuclear factor E2-related factor-2 (NRF2) by a direct physical interaction, leading to induction of NRF2 target gene products, such as heme oxygenase 1 (HO1), cytochrome P450 3A4 (CYP3A4), glutathione S-transferase A2 (GSTA2), glutathione S-transferase P1 (GSTP1), multidrug resistance protein 1 (MDR1), multidrug resistance-associated protein 1 (MRP1), MRP4, and MRP5 [154]. NRF2 is a master transcription factor that regulates the expression of genes in oxidative stress responses, detoxification, and drug resistance [155].

Although HER3 has a catalytically inactive kinase domain, it serves as a signaling platform by heterodimerizing other receptors [156]. Overexpression of HER3 or its ligand heregulin (HRG, also known as neuregulin-1 (NRG1)), has been linked to EGFR TKI resistance [157,158,159]. Additionally, an increase in HER3 was identified in patients with TNBC treated with cetuximab or panitumumab [159]. A compensatory increase in and activation of HER3 has been identified as a result of gefitinib treatment in breast cancer cells through release of the AKT-mediated negative feedback loop [157]. Since AKT negatively regulates Forkhead box O (FOXO) transcription factors that are responsible for transcription of RTKs, such as HER3, IGF1R, FGFR2, and insulin receptor, blocking the PI3K/AKT pathway results in the expression of these RTKs in TNBC cells [160]. Activated protein–tyrosine kinase 2 (PYK2 or focal adhesion kinase 2 (FAK2)) has also been reported to inhibit the binding of HER3 to neural precursor cell expressed developmentally downregulated protein 4 (NEDD4) [161]. Since NEDD4 is an E2 ubiquitin ligase for HER3, disruption of the HER3–NEDD4 interaction leads to blocking of the proteasome-dependent degradation of HER3. In addition, EGFR inhibition induced upregulation of HER3, leading to EGFRi resistance, and increased HER3 was associated with a decreased response of patients with TNBC to panitumumab or cetuximab in clinical studies [159].

Activated AXL has also been linked to intrinsic resistance to osimertinib [162,163]. Overexpression of AXL has been associated with acquired resistance to EGFRis in NSCLC [164,165]. In the TNBC cell line MDA-MB-231, AXL was found to be overexpressed and physically associated with EGFR [166]. Additionally, *AXL* knockdown (KD) reduced the EGFR-dependent activation of downstream signaling and the EGF-stimulated migration of MDA-MB-231 cells.

Adaptive kinome reprogramming has been established as a mechanism of kinase inhibitor resistance via upregulation of expression of different RTKs [167]. For example, blocking the RAS/RAF/MEK/ERK pathway may lead to destabilization of the oncogene product v-myc avian myelocytomatosis viral oncogene homolog (MYC) through a proteasome-dependent manner in TNBC cells (see Section 3.3.1. Activation of the RAS/RAF/MEK/ERK pathway.) [168,169]. Since *MYC*-KD induces the expression of the platelet-derived growth factor receptor beta (*PDGFRβ*), *VEGFR2*, and platelet-derived growth factor subunit B (*PDGFB*) and increased tyrosine phosphorylation of PDGFRβ, VEGFR2, HER3, and AXL in the TNBC cell line SUM159PT [168], the degradation of MYC by kinase inhibition causes resistance of cancer cells to the inhibition.

### 3.3. Activation of Downstream Pathways

The components in the downstream pathways of RTKs, such as the RAS/RAF/MEK/ERK, PI3K/AKT/mTORC1, and JAK/STAT3 pathways, have been studied as intervening nodes either for monotherapy or combination therapy for TNBC cells [170,171,172,173]. However, the EGF-induced activation of the RAS/RAF/MEK/ERK and PI3K/AKT/mTORC1 pathways persists even in the presence of lapatinib in EGFRi-resistant TNBC cell lines, suggesting that EGFRi resistance occurs through the bypassing activation of downstream survival pathways [99].

#### 3.3.1. Activation of the RAS/RAF/MEK/ERK Pathway

Single nucleotide point mutations in codons 12 and 13 of *KRAS* lead to amino acid substitutions including G12D, G12V, G12C, G12S, G12A, and G13D [36]. All of these mutations impair KRAS GTPase activity, causing accumulation of the hyperactive GTP-bound form of KRAS proteins [174]. This accumulation of active KRAS results in the EGFR-independent constitutive activation of the RAS/RAF/MEK/ERK pathway.

Interestingly, only a small proportion of breast cancers have mutations in *KRAS* (5%), *HRAS* (1%), and *BRAF* (2%) [175]. Nevertheless, hyperactivation of the RAS/RAF/MEK/ERK pathway is associated with TNBC [176]. However, its clinical relevance remains to be determined. The 3′UTR, which contains a polymorphism in a *let-7* miRNA complementary site of a *KRAS* variant has been linked to TNBC clinical samples with altered gene expression [177]. This variant was further associated with a low level of *let-7* expression in TNBC. Interestingly, a recent study demonstrated that TNBC has higher KRAS signaling than ER-positive breast cancer [178]. In addition, patients with TNBC with enriched KRAS signaling gene sets are associated with inflammation and a favorable tumor-immune microenvironement, as well as improved DFS and OS [178].

Aberrant activation of the RAS/RAF/MEK/ERK pathway may induce stabilization of MYC via ERK-dependent phosphorylation at S62 in TNBC cells [168]. Thus, p-MYC could escape proteasomal degradation [169]. Furthermore, the RAS-mediated activation of the PI3K pathway leads to blocking of the inhibitory phosphorylation of MYC at T58 by GSK3β, which is negatively regulated by AKT. In addition, the phosphorylation and stability of the beta-transducin repeat-containing protein 1 (β-TrCP1), which antagonizes the E3 ubiquitin ligase FBXW7 to stabilize MYC [179], is also regulated by the PI3K/mTORC2 pathway in TNBC cells [171]. Overexpression of MYC has been reported in TNBC and is associated with drug resistance [180,181,182]. β-TrCP1 is also a F-box/WD repeat-containing protein (FBXW) subfamily member and the substrate-recognition subunit of the SKP1-cullin 1-F-box protein (SCF) ubiquitin–ligase complex [183,184,185]. β-TrCP1 plays roles as a both an oncogene and a tumor suppressor in a tissue-specific and cellular-context-dependent manner. *β-TrCP1*-KD has been reported to suppress the growth of the MSL TNBC cell lines HS578T and MDA-MB-231 [171]. Overexpression of β-TrCP is known to promote tumorigenesis and is associated with various cancers such as breast, colorectal, gastric, hepatoblastoma, melanoma, pancreatic, and prostate cancers [184,185]. β-TrCP binds to phosphorylated DEP-domain-containing mTOR-interacting protein (DEPTO), a negative regulator of mTORC1/2, leading to degradation of DEPTOR [186,187,188]. In contrast, *β-TrCP*-KD results in DEPTOR accumulation, which causes reduced mTOR and S6K activities and autophagy induction [187,188].

#### 3.3.2. Activation of the PI3K/AKT/mTORC1 Pathway

The PI3K/AKT/mTORC1 pathway is a major effector of the EGFR. Three hotspot *PIK3CA* (PI3K catalytic subunit)-activating mutations include E453K, E545K, and H1047R [189]. Mutation analyses of TNBC have found frequent mutations in *PIK3CA* (~10% to 24%) and phosphatase and tensin homolog (*PTEN*) (~8%) [190,191]. In addition, high frequencies of *PTEN* loss or inactivation (35%) and *AKT3* amplification (28%) were also found in TNBC [192]. PTEN is a lipid phosphatase that inhibits the activity of PI3K by removing a phosphate from phosphatidylinositol (3,4,5) triphosphate to form phosphatidylinositol (4,5) bisphosphate [193]. PTEN serves as a tumor suppressor, and loss of PTEN activates the PI3K/AKT/mTORC1 pathway [194]. In fact, the PI3K/AKT/mTOR pathway has been reported to be activated in approximately 60% of patients with TNBC [167].

*PIK3CA* mutations have also been associated with resistance of TNBC cell lines to chemotherapy [195]. In addition, activating *PIK3CA* mutations in basal-like breast cancer were found to induce paracrine activation of AREG/EGFR/ERK signaling [196]. High levels of both AREG mRNA and protein were correlated with the mutation status of *PIK3CA* in breast cancer cell lines.

Among three isoforms (AKT1, AKT2, and AKT3), AKT3 has been identified as overexpressed both at the DNA and mRNA levels in TNBC [197]. In addition, high levels of p-AKT (T308) and p-AKT (S473), markers for the PI3K/AKT activation, are associated with TNBC [192].

#### 3.3.3. Activation of the NF-κB Pathway

Aberrant activation of the nuclear factor kappa light chain enhancer of activated B cells (NF-κB) pathway also confers intrinsic and acquired resistance [198]. Furthermore, NF-κB is constitutively active in many type of cancers [199]. The activity of NF-κB is negatively regulated by inhibitor of NF-κB (IκB) through a complex formation. The dissociation of IκB is controlled by the IκB kinase (IKK) complex-induced phosphorylation and subsequent degradation of p-IκB [199,200,201]. EGFR-NF-κB crosstalk in cancer cells has previously been identified, showing that (1) EGFR directly or indirectly activates NF-κB in human various cells, including ER-negative breast cancer cells [202,203,204,205,206,207,208,209,210], while (2) the IKK/NF-κB pathway activates EGFR signaling [211,212,213,214]. In addition, activated NF-κB confers EGFRi resistance. Inhibition of NF-κB consistently sensitizes cancer cells to EGFRis [202,215,216,217,218,219]. For example, NF-κB activates the expression of the anti-apoptotic proteins BCL2-like protein 1 isoform 1 (BCL2L1, also known as BCL-xL) and BCL2-related protein A1 (BCL2A1) in a Mucin 1 carboxy terminal subunit (MUC1-C)-dependent manner [220,221]. Notably, a high expression level of MUC1-C has been reported in TNBC [222,223]. Although the IKK/NF-κB pathway has been recognized as a potential therapeutic target for TNBC treatment [209,217,224], further studies are needed to determine the effects of IKK/NF-κB inhibition on EGFRi resistance in TNBC. Our group very recently identified an IKK inhibitor as a sensitizer of EGFRi in TNBC cells (You et al., manuscript in preparation).

#### 3.3.4. Activation of the c-Jun N-Terminal Kinase (JNK) Pathway

The activation of JNKs has been correlated with EGFR expression in TNBC [225] and leads to increased *c-Jun* mRNA associated with a decreased DFS among patients with TNBC [226]. The JNK pathway has been linked to increases in invasiveness, angiogenesis, and metastasis; cancer stem cell (CSC) phenotype; and acquired resistance to EGFR/HER2-targeted therapies [226,227,228,229,230]. Of interest, the EGFR and HER2 dual inhibitor lapatinib has been reported to induce increased p-c-Jun and p-JNK in the TNBC cell line MDA-MB-231, resulting in resistance of these cells to lapatinib [231] (see Section 4.1.6. Combination with JAK Inhibitors).

#### 3.3.5. Activation of the Notch Pathway

The Notch signaling pathway is composed of single-pass transmembrane Notch receptors in the signal receiving cells and their ligands jagged and delta-like proteins in the signal sensing cells [232,233]. Dysregulation or activation of the Notch signaling pathway is a hallmark for TNBC, with a strong correlation with aggressive clinicopathological features [232]. Four Notch receptors have been associated with tumor growth (Notch1, Notch2, and Notch3), CSC regulation (Notch1, Notch2, Notch4), tumor invasion and metastasis (Notch1, Notch2, Notch3, Notch4), angiogenesis (Notch3), and drug resistance (Notch1 and Notch3) [232,233]. Inactivation of Notch3, either by knockdown or by raft depletion, has been reported to sensitize the TNBC cell lines BT549 and MDA-MB-468 to gefitinib by enhancing dephosphorylation of EGFR at Y1173 and its intracellular retention, preventing its membrane localization into lipid rafts [234]. The dephosphorylation of p-EGFR (Y1173) is promoted by the *Notch3*-KD-induced interaction of EGFR with the protein tyrosine phosphatase H1 (PTPH1). These results suggest that reducing the EGFR cell surface expression may prevent survival signaling to enhance drug-induced cell death [234].

### 3.4. Others Resistance Mechanisms

#### 3.4.1. Expression of Mutant p53 (mtp53)

Mutations of the tumor suppressor *p53* are well established as being associated with approximately 50% of human cancers [235,236,237,238,239]. A panel of TNBC cell lines has been identified to express mutant p53 (mtp53) [240], which is commonly found in TNBC [120,192]. For example, one study reported that 297 of 450 (66.0%) breast cancer clinical samples contained *p53* mutations. More frequent *p53* mutations were found in TNBC (74.8%) than in HER2-positive breast cancer (55.4%) [241]. In addition, a separate study conducted a mutation analysis of 104 primary TNBC cases and revealed that approximately 85% had somatic *p53* mutations [190]. Mutations of *p53* often result in stabilization and overexpression of mtp53 protein [240], and overexpression of mtp53 has been associated with cancer resistance to anticancer drugs [242,243].

One plausible mechanism of EGFR overexpression in TNBC is the oncogenic mtp53 [244,245,246]. The activity of EGFR is regulated at multiple levels, including endosomal recycling. Endocytosis occurs to remove part of the plasma membrane including associated proteins, such as EGFR, and subsequently to form internalized membrane vesicles (early endosomes). It is a very active process, such that the entire plasma membrane is endocytosed at least once per hour [247]. Upon ligand binding, the ligand–EGFR complex is internalized by endocytosis and the early endosome forms multivesicular bodies (MVBs). MBVs now may follow three alternative pathways: (1) fuse with the lysosome to degrade their cargo; (2) go back to the plasma membrane to recycle their cargo; (3) fuse with the plasma membrane to release intralumenal vesicles into the extracellular space as exosomes [248,249,250]. P53^R273H^ enhances recycling of EGFR to the plasma membrane in combination with integrin α5β1, leading to constitutive activation of EGFR–integrin signaling [251]. Mtp53 indirectly promotes the interaction of the Rab coupling protein (RCP) with α5β1 in the EGFR–integrin complex, while the presence of RCP has been associated with enhanced recycling of the receptor complex [251,252].

In addition, mtp53 upregulates *EGFR* expression through suppression and upregulation of miR-27a and miR-155, respectively [245,246]. Furthermore, p53^R273H^ has been demonstrated to bind and suppress the promoter of miR-27a, which targets the 3′-UTR of *EGFR* [246]. In the TNBC cell line MDA-MB-468, stable expression of miR-27a mimics or p53 shRNA reduced the EGFR levels, with decreased colony formation in vitro and reduced the volume of xenograft tumors in vivo. The mtp53s, including R248Q, R282W, and R249S, upregulate the expression of miR-155 by relieving p63-mediated transcriptional suppression [245]. MiR-155 targeted the ZNF652, a transcriptional repression of *EGFR*. The ZNF652 level was consistently reduced and inversely correlated with the miR-155 level in TNBC cell lines with mtp53s.

#### 3.4.2. Overexpression of Anti-apoptotic Proteins

Upregulation of anti-apoptotic proteins confers drug resistance in many cancers [253,254,255]. Two major anti-apoptotic protein family members tightly control both the intrinsic and extrinsic apoptosis pathways: (1) inhibitors of apoptosis proteins (IAPs) control the activity of caspases; (2) B-cell lymphoma 2 (BCL2) family proteins regulate the integrity of the mitochondrial outer membrane [253].

The anti-apoptotic members of BCL2 family proteins include BCL2, BCL2L1 (BCL-xL), BCL2-like 2 (BCL2L2, BCL-w), and myeloid cell leukemia 1 (MCL1) [256]. High expression levels of BCL2 are also associated with a poor clinical prognosis of TNBC [257]. A human tumor microarray analysis demonstrated that TNBC tumors express EGFR and co-express BCL-xL or both BCL-xL and BCL2 [258]. MCL1 has been determined to be commonly overexpressed in TNBC and is associated with a poor clinical prognosis. MCL1 is further stabilized by the overexpressed MUC1-C-mediated activation of the RAS/MEK/ERK and PI3K/AKT pathways in TNBC cells [259]. MUC1-C has additionally been determined to activate the transcription of *BCL2A1* in an NF-κB-dependent manner in TNBC cells [220]. Recently, upregulation of MCL1 by elongator (ELP) complex has been reported to mediate resistance to the EGFRi erlotinib [260]. Depletion of ELP proteins, such as ELP3, ELP4, ELP5, and ELP6, sensitized TNBC cells to erlotinib, while *ELP4*-KD reduced expression of MCL1 in the TNBC cells in the presence of erlotinib.

#### 3.4.3. Contributions of Phosphatases

In human genomes, 189 known and predicted protein phosphatases have been identified [261]. Protein phosphatases antagonize the action of protein kinases by hydrolysis of phosphate groups from target proteins. Both protein kinases and phosphatases work as key regulators of various cellular processes in normal and disease conditions. Limited number of studies reported the potential roles of protein phosphatases in EGFRi resistance in TNBC.

The protein tyrosine phosphatase non-receptor type 12 (PTPN12) has been known as a tumor suppressor in TNBC [262]. Loss of PTPN12 function is frequently found in human TNBC, leading to activation of HER2, EGFR, and PDGFRβ pathways. These results suggest a rationale to target multiple RTK pathways in TNBC [262]. In addition, the activation or compensation of PTPN12 activity may also overcome EGFRi resistance in TNBC.

The SRC homology region 2-containing protein tyrosine phosphatase (SHP2; also known as PTPN11) has been demonstrated to contribute adaptive resistance to ERK signaling inhibition in TNBC [263]. In fact, SHP2 regulates both upstream and downstream targets of RTKs, including EGFR [264]. Silencing of SHP2 by shRNA suppressed the RAS/RAF/MEK/ERK and PI3K/AKT pathways in TNBC cells. In addition, SHP2 depletion reduced expression of RTKs such as EGFR, FGFR, and MET, leading to suppression of TNBC cell proliferation, anchorage-independent growth, and mammosphere formation. Although small molecule inhibitors of SHP2 have been developed as potential therapeutics for treatment of cancers including TNBC [265,266,267], the efficacy of the combination of these inhibitors with EGFRi remains to be determined.

#### 3.4.4. Overexpression of the Heat Shock Protein 90 (HSP90)

As a molecular chaperone, HSP90 interacts with and regulates the stability and function of over 200 client proteins, including EGFR, HER2, ALK, MET, and AKT [268,269,270,271]. Since overexpression of HSP90 has been evidenced in cancers, including breast and cervical cancers and osteosarcoma, HSP90 inhibitors (HSP90is) show therapeutic effects by suppressing multiple oncogenic pathways that are activated by its client proteins. For example, the HSP90i geldanamycin reduced the level of RPS6 in a proteasome-dependent manner [272]. Notably, *RPS6*-KD suppressed the proliferation of TNBC cells over time [15]. However, the efficacy of HSP90is has been limited in patients with TNBC due to both intrinsic and acquired resistance [273]. To the best of our knowledge, the potential of combined treatment of HSP90is and EGFRis has not been tested yet.

#### 3.4.5. Overexpression of the Estrogen Receptor Alpha (ERα) Variant

A novel variant of ERα with a molecular weight of 36 kDa (ERα36 or ER36) has been identified [274]. ERα36 is an alternatively spliced product of *ERα46* that is transcribed by an alternative promoter located in the first intron of the *ERα66* gene. ERα36 differs from the canonical ERα66, as it lacks both transcription activation domains (activation factor (AF)-1 and AF-2) but retains the DNA-binding domain and partial ligand-binding domain. ERα36 is involved in mammary tumor progression and resistance to drug treatment due to its ability to activate non-genomic signaling pathways such as the PI3K/AKT/mTORC1 and RAS/RAF/MEK/ERK pathways [275,276]. The primary location of ERα36 is the cytoplasm, in which it is associated with the plasma membrane to induce signal transduction [277]. A positive feedback loop of ERα36/EGFR has been identified in TNBC cells: (1) in response to estrogen, ERα36 physically associates with the EGFR/v-src avian sarcoma (Schmidt-Ruppin A-2) viral oncogene homolog (SRC)–SRC homology 2 domain-containing-transforming protein C (SHC) complex to induce phosphorylation of EGFR (Y845) and SRC (Y416); (2) EGFR-induced signaling activates the transcription of *ERα36* through an AP-1 site in its promoter; (3) ERα36 prevents EGFR protein from proteasome-dependent degradation [278]. In addition, tamoxifen, a selective ER modulator [279], has been identified to enhance the stemness and metastasis of breast cancer by upregulating aldehyde dehydrogenase 1A1 (*ALDH1A1*) transcription in cancer cells through direct binding to and activation of ERα36 [280].

#### 3.4.6. Overexpression of Adenosine Diphosphate Ribosylation Factor (ARF)

ARF1 has been associated with resistance to anticancer drugs in various cancers [281,282,283,284,285]. ARF1 is a member among 29 human ARF family members that belong to the small GTPase RAS superfamily [286,287]. GTP binding to ARF1 switches its conformation to an active one from the GDP-bound inactive form [286,287]. ARF1 is a key regulator that maintains the structure and function of the Golgi apparatus. Recently, ARF1 overexpression has been reported to promote resistance of TNBC cells to the EGFRi gefitinib [288]. Of interest, treatment of gefitinib to MDA-MB-231 cells induces increased ARF1 activity through its enhanced recruitment of AXL. ARF1 is also involved in the gefitinib-induced activation of ERK1/2, AKT, and SRC and inhibits gefitinib-induced EGFR internalization and subsequent degradation. Pharmacological inhibition of ARF1 activity potentiates gefitinib-mediated anticancer activity in MDA-MB-231 cells [288] (see Section 4.2.4. Combination with ARF Exchange Activity Inhibitors (ARFis)).

#### 3.4.7. Dysregulation of Reactive Oxygen Species (ROS)

ROS are important intracellular second messengers [289], and dysregulation of ROS is associated with anticancer drug resistance [290,291,292]. Specifically, ROS inactivate SHP2 by transient oxidation of the catalytic cysteine residue, leading to EGFR transactivation [293]. ROS also contribute to anticancer drug resistance through metabolic deregulations, including increased drug efflux, altered oncogenes or tumor suppressors, drug inactivation, epithelial–mesenchymal transition (EMT), and enhanced DNA repair [292].

#### 3.4.8. Expression of the Cluster of Differentiation 44 (CD44)

CD44 is a type 1 transmembrane glycoprotein that serves as a receptor for hyaluronic acid [294,295,296], type 2 and IV collagens [297,298], E-selectin [299,300], fibronectin [301], matrix metalloproteinase 9 (MMP9) [302], and osteopontin (OPN) [303]. CD44 is also known as extracellular matrix receptor type 3 (ECM-III) [304], Hermes antigen [305], homing cell adhesion molecule (HCAM) [306], lymphocyte homing receptor [307], and phagocytic glycoprotein-1 (Pgp-1) [304]. The biology of CD44 is extremely complex, and more than 29 isoforms from a single gene consisting of 19 exons have been described in humans [308]. The shortest standard form (CD44s) is encoded by the 10 constant exons, whereas CD44 variant isoforms (CD44v) are encoded by alternatively spliced mRNAs containing the 10 constant exons and any combination of an additional 9 variant exons [309].

CD44 is involved not only in tissue tropism of stem cells and their exosomes [248,310,311,312], but also in tumor initiation and progression [313]. CD44 has also been established as a potential CSC marker that contributes to cancer proliferation, metastasis, and drug resistance [314]. The expression of CD44 has been correlated with the EGFR level in a variety of cancer, and their expression is positively regulated reciprocally [315]. CD44 also serves as a co-receptor for RTKs to stimulate downstream signaling pathways [309]. Recently, siRNA-based *CD44s*-KD has been reported to sensitize TNBC cells to cetuximab, with enhanced EGFR degradation and downregulation of p-AKT and p-ERK [316].

In addition, OPN has been identified as a biomarker for predicting responsiveness to the EGFR TKI, erlotinib, in two TNBC cell lines, MDA-MB-231 and MDA-MB-468 [317]. Engineered cell lines that overexpress OPN are more sensitive to erlotinib-mediated growth inhibition than parental cells. This might be dependent on OPN binding to integrin and provoking activation of EGFR kinase activity [318]. Consistent with these findings, OPN has been associated with poor outcomes among patients with breast cancer [319,320,321,322,323].

#### 3.4.9. Overexpression of Annexin A2 (ANAX2)

ANXA2 is a member of the annexin family of calcium-dependent phospholipid binding proteins containing an annexin repeat [324,325]. ANXA2 has been reported to play an important role in cancer cell proliferation, metastasis, and drug resistance [326]. It also contributes to EGFR dimerization and endocytic recycling [327,328]. Recently, knockdown of *ANXA2* in the TNBC cell line MDA-MB-231 has been shown to increase gefitinib sensitivity [324]. In contrast, overexpression of ANXA2 induces resistance to gefitinib in MCF7 breast cancer cells. Phosphorylation of ANXA at Y23 has been further negatively correlated with gefitinib sensitivity in TNBC cells [324].

#### 3.4.10. Induction of Autophagy

Autophagy is the controlled removal of unnecessary or dysfunctional components from cells, enabling the recycling of cellular components [329]. Although autophagy has tumor-suppressive roles in normal cells by removing damaged organelles [330], it also protects cancer cells from cell death [331,332]. Mounting evidence further supports the idea that drug-induced autophagy causes resistance of cancer cells, and combined inhibition of autophagy is a plausible strategy to overcome this resistance [331,332,333].

EGFR TKIs and neutralizing antibodies have been shown to induce autophagy in cancer cells [334,335,336,337]. In addition, the EGFR TIK-induced accumulation of the inactive EGFR in endosomes may play a role in autophagy induced by EGFR TKIs [338]. However, more details remain to be determined to understand the function of autophagy in cancer. A recent study suggested that EGFRi-induced autophagy has a pro-survival role in TNBC cells, while combined inhibition of autophagy with EGFRis confers anticancer effects in TNBC cells [339] (see Section 4.1.11. Combination with Rho-Associated, Coiled-Coil-Containing Protein Kinase (ROCK) Inhibitors).

#### 3.4.11. Contribution of Secretomes

Secretomes, including cytokines, growth factors, extracellular nucleic acids, and extracellular vesicles (EVs), either from cancer cells or from stromal cells, confer drug resistance [147,340,341,342,343]. For example, HGF was identified as a resistant growth factor in a co-culture screening of 23 stromal cell types with 45 cancer cell lines against 35 anticancer drugs [344]. NRG1 was also shown to drive the resistance of cancer cells to PKIs [147].

IL-25 (IL-17E), a member of the IL-17 family, binds and activates the IL-17RA/IL17RB heterodimer through direct biding to IL-17B [345,346]. The IL-25/IL17RB pathway has contributed to the resistance of TNBC to EGFRis [347]. IL-25 activates the EGFR pathway in TNBC cells with resistance to the EGFRi gefitinib. IL-25 also activates PYK2 and SRC, leading to the SRC-dependent activation and nuclear translocation of p-STAT3 and p-EGFR [347].

EVs collectively refer to the membranous vesicles released from living cells, such as exosomes, microvesicles, and apoptotic bodies [248,348]. Tumor-derived EVs have been demonstrated to mediate drug resistance via multiple mechanisms. For example, EVs can: (1) reduce the accumulation of anticancer drugs inside cancer cells; (2) traffic functional cargo, activating pro-survival or anti-apoptotic signals in cancer cells; (3) suppress immune reactions in cancer microenvironments; (4) enhance EMT and induce CSC-like properties (reviewed in [349]). In addition, tumor-derived EVs from patients’ body fluids could be used for liquid biopsy to diagnose disease status and drug sensitivity [350]. More importantly, tumor-derived EVs may serve as therapeutic targets, drug delivery vehicles, or therapeutics themselves [350].

Taken together, the understanding of the complicated interplay of autocrine and paracrine signaling occurring between tumor cells and normal cells in their associated microenvironment will provide valuable information to develop more effective clinical strategies to combat drug resistance.

## 4. Combination Strategy for Overcoming EGFRi Resistance in TNBC

Combination therapies have been proven to overcome resistance to EGFR TKIs in clinical settings for NSCLC [351,352,353,354,355,356,357,358,359,360,361,362,363]. A variety of combination strategies has been explored preclinically in TNBC cells (Table 3). Among the 73 publications analyzed, 60 reported the preclinical results of combinatorial therapies. Combination approaches were explored as early as 2004 with small-molecule EGFR TKIs and the anti-EGFR antibodies [364]. with a growing number of small-molecule PKIs, combination strategies have been applied to target multiple cellular pathways. We briefly summarize EGFR-potentiating strategies according to the target categories.

### 4.1. PKIs

Protein kinases play pivotal roles in the pathogenesis of various diseases, including cancer. A plethora of PKIs targeting such kinases are under development to be used for therapeutic interventions. As of 23 December 2020, sixty-two PKIs have already been approved by the US FDA [49]. In addition, three more PKIs, including tepotinib (Merck), trilaciclib (G1 Therapeutics), and umbralisib (TG Therapeutics), have been approved by the US FDA as on 2 March 2021(PKIDB: a curated, annotated, and updated database of protein kinase inhibitors in clinical trials, https://www.icoa.fr/pkidb/, accessed on 2 March 2021) [457,458]. The FDA-approved PKIs that have been tested for efficacy in combination treatment with EGFRis in TNBC cells are shown in Table 4. Notably, most anti-EGFR therapeutics used in combination are also FDA-approved EGFR TKIs or antibodies.

#### 4.1.1. Combination with Other EGFRis

Interestingly, among 60 preclinical studies, no study has reported on the combination of different generation EGFR TKIs in TNBC (Table 3). As mentioned earlier (see Section 3.1. EGFR Mutations or Amplification), this may reflect the fact that TNBC has been reported to possess rare *EGFR* mutations.

Another interesting strategy is the combination of small-molecule EGFRis and anti-EGFR antibodies. Since EGFRis target the intracellular kinase domain and anti-EGFR antibodies bind to the ectodomain, the mode of action would be complementary [364,403] This hypothesis has been proven in several studies. A combination of gefitinib with cetuximab was evaluated across various cancer cell lines, including breast, colon, prostate, and vulvar squamous carcinoma cells [364]. A TNBC cell line MDA-MB-468 was demonstrated to be a susceptible cell line in response to the gefitinib and cetuximab combination, whereas MDA-MB-435S did not respond to this combination [364]. MDA-MB-435S has been shown to express no detectable EGFR [459]. A combination of cetuximab or panitumumab with erlotinib or gefitinib has shown an anticancer effect in TNBC cell lines that harbor *EGFR* amplification (MDA-MB-468) and wild-type *KRAS* and *PTEN* (SUM1315MO2) [403]. This combination induced G_1_ cell cycle arrest and subsequent apoptosis with concomitant inhibition of the RAS/RAF/MEK/ERK pathway. Susceptible TNBC cells were sensitive to anti-EGFR antibody alone, while TNBC cells containing mutations in *KRAS* (MDA-MB-231) or *PTEN* (HCC1937) were resistant to anti-EGFR antibody treatment. Consistent with these results, anti-EGFR antibody alone could not inhibit the RAS/RAF/MEK/ERK pathway in resistant cells [403].

#### 4.1.2. Combination with MET Inhibitors (METis)

As mentioned, MET activation confers resistance to EGFRis in TNBC cells [152,153] (see Section 3.2. Activation of Bypass Signaling Pathways). An earlier study demonstrated that the addition of the METi tepotinib reduces resistance of MDA-MB-468 cells to gefitinib [153,424]. Interestingly, the gefitinib and tepotinib treatment together reduced cell growth of MDA-MB-468 cells but not that of MDA-MB-231 cells, whereas the same combination reduced colony formation of both cell lines. Furthermore, the addition of cetuximab to tepotinib had no effect on MDA-MB-231 colony formation but had an effect on MDA-MB-468 colony formation. Mechanistically, the gefitinib and tepotinib treatment synergistically reduced levels of p-AKT (T308), p-ERK (T202/Y204), and p-ribosomal protein S6 (p-RPS6) (S235/236) in MDA-MB-468 cells, while no significant suppression was observed by single-agent treatment [153]. More interestingly, signal activation, including p-MET, p-EGFR, p-HER2, p-AKT, and p-ERK signaling, was provoked by EGF and HGF treatment of susceptible cells (MDA-MB-468) but not of non-susceptible cells (MDA-MB-231 and T47D). Antibody microarray analysis identified that p-MET and p-HER3, which were induced by EGF and HGF co-treatment, were abolished by the gefitinib and tepotinib combination in MDA-MB-468 cells. In addition, knockdown of *EGFR* combined with the METi PHA-665752 or the erlotinib and PHA-665752 combination synergistically reduced the cell viability of TNBC cells in vitro [150].

The MSL subtype TNBC cell lines HS578T and MDA-MB-231 have been reported to be more resistant to gefitinib than BL subtype cell lines such as MDA-MB-468 and SUM149PT [15,378]. The addition of the METi SU11274 potentiated the cytotoxicity of gefitinib and reduced X-linked inhibitor of apoptosis (XIAP). Most interestingly, the gefitinib and SU11274 treatment markedly reduced the levels of both total RPS6 and p-RPS6 (S235/236). The reduction in the total RPS6 occurred as early as 2 h and was sustained over time to 16 h post-treatment, whereas the reduction in p-AKT (S473) returned to normal levels at 24 h post-treatment. Furthermore, knockdown or *RPS6* alone reduced proliferation of HS578T and MDA-MB-231 TNBC cells [15]. The mechanism of RPS6 reduction achieved by this combination remains to be determined.

A combination of METis and EGFRis was further evaluated with the METis glexatinib (MGCD265) and crizotinib (PF-02341066) and the EGFRi erlotinib [400]. Glexatinib and crizotinib are multitarget, ATP-competitive inhibitors of DDR2, AXL, MERTK, PDGFRα, VEGFR3 (FLT4), FLT3, MET, VEGFR2 (KDR), and PDGFRβ [399]; and of ROS1, ALK, LTK, FER, FES, MET, PTK2B (FAK2), TNK2, PTK2 (FAK), TRKA/B/C, and FRK (PTK4), respectively [401,402]. Interestingly, crizotinib also inhibits EGFR^L858R/T790M^ and EGFR^T790M^ [402]. The addition of glexatinib or crizotinib to erlotinib synergistically reduced tumor growth in a patient-derived xenograft (PDX) model with consistent reduction in p-ERK1/2 and p-RPS6 in PDX tumor samples [400].

The MET and VEGFR inhibitor foretinib (XL880) [422] has been identified as a potentiator of the dual EGFR and HER2 inhibitor lapatinib in reducing the viability and migration of TNBC cells [423]. Furthermore, the invasiveness of TNBC cells was reduced by the lapatinib and foretinib combination, with robust reductions in the number of invadopodia formed and the ability of gelatin digestion. This combination could reduce p-AKT (S473) in BT549 and MDA-MB-231 cells but not p-ERK1/2 (T202/Y204) in MDA-MB-231 cells. Further study is needed to determine the molecular mechanism of this combination effect.

#### 4.1.3. Combination with PI3K/AKT/mTORC1 Inhibitors

The first report on the potentiation of EGFRis via vertical inhibition of the PI3K/AKT/mTORC1 pathway demonstrated that PI-103 enhanced apoptotic cell death in the BL subtype TNBC cell lines MDA-MB-468 and SUM149PT [378]. No potentiation was observed in the MSL TNBC cell lines HS578T and MDA-MB-231. Synergistic reduction of anti-apoptotic proteins, including XIAP, BCL2, and MCL1, was detected in sensitive cells. Overexpression of these anti-apoptotic proteins is associated with drug resistance and a poor prognosis [253] (See Section 3.4.2. Overexpression of Anti-apoptotic Proteins). For example, MCL1 is regulated at multiple levels in cancer cells. Transcription factors such as STAT3 and MYC activate *MCL1* transcription [460]. In addition, inhibitory phosphorylation by AKT alleviates the GSK3β-mediated destabilization of MCL1. AKT further promotes the stability of MCL1 through its phosphorylation by mTORC1. A chemically modified LY294002 with the RGDS integrin-targeting peptide was also reported to potentiate gefitinib [461] in MDA-MB-468 TNBC cells [460]. The peptide leads to targeting of the RGDS integrins αvβ3 and α5β1 and is released to liberate LY294002 by hydrolysis at physiological pH [462].

Dual blocking of EGFR and PI3Kβ has been identified as an effective way to overcome the compensatory activation of the EGFR downstream signaling in a G protein-coupled receptor of thrombin (PAR1/F2R)-dependent manner [451]. A genome-wide shRNA screening approach has identified EGFR as an additional target that synergizes with PI3K inhibitors and AKT inhibitors in the *PTEN*-deficient TNBC cell line MDA-MB-468. Pharmacological inhibition of EGFR by gefitinib in combination with AZD8186, which is a PI3K inhibitor that specifically targets PI3Kβ, PI3Kδ, and PI3Kα (Table 3) [452], synergistically reduces cell proliferation of *PTEN*-null TNBC cells but not that of *PTEN* wild-type TNBC cells [451]. This synergism was confirmed in vivo in orthotopic xenograft models of HCC70 or MDA-MB-468 cells by the erlotinib and AZD8186 combination. In addition, this combination also showed anticancer effects in an immunocompetent syngeneic mouse model. Among the components in the PI3K/AKT/mTORC1 pathway, the response of p-RPS6 (S235/236) represents a useful predictive marker for sensitivity to PI3K inhibition. A co-immunoprecipitation assay revealed that PI3Kβ is a key component of the activated PI3K/AKT/mTORC1 pathway in *PTEN*-null TNBC cells. Targeted deletion screening by CRISPR-Cas9 further identified that targeting G protein β and γ subunits sensitized TNBC cells to EGFRis and PI3Kis. Inhibiting G protein β and γ subunits blocked the PAR1-dependent activation of AKT. Vorapaxar, an inhibitor of PAR1, consistently synergizes the effect of pictilisib or lapatinib to reduce colony formation of MDA-MB-468 cells [451].

Rapamycin (silorimus) is a specific mTOR inhibitor that directly inhibits mTORC1 [463,464]. Rapamycin, in combination with lapatinib, has been reported to reduce the increase in xenograft tumors of MDA-MB-231 and MDA-MB-468 cells [368]. The lapatinib and rapamycin combination preferentially induces apoptosis in MDA-MB-468 cells compared to MDA-MB-231 cells both in vitro and in vivo. Similar to previous reports [465,466], the level of p-eIF4E was associated with apoptotic resistance of MDA-MB-231 cells [368].

Analogs of rapamycin (rapalogs), including temsirolimus, everolimus, and deforolimus, have been developed as mTORC1 inhibitors [467]. A separate study reported that the blocking of mTOR by temsirolimus in the presence of gefitinib reduces cell growth, viability, colony-forming ability, and cap-dependent translation, with concomitant decreases in p-eIF4B in the TNBC cell lines BT20, MDA-MB-231, and MDA-MB-468 [387]. Similar to RPS6 [468], eIF4B is a converging point of the RAS/RAF/MEK/ERK and PI3K/AKT/mTORC1 pathways [469]. Interestingly, p-p90RSK (S380) rather than p70S6K (T389) was found to be a mediator of this combination effect in these TNBC cells.

When combined with the EGFRi, AEE788 rapalogs induce synergistic reduction of TNBC cell proliferation [430]. An antiproliferation screening of 378 small-molecule PKIs in combination with rapamycin was performed against 19 TNBC cell lines. Multiple EGFRis, such as AEE788, afatinib, AC480, AZD8931, AZD9291, AST-1306, and gefitinib, have been shown to induce antiproliferative effects synergistically or additively in combination with rapamycin in the TNBC cell line SUM149PT. AEE788 is a dual inhibitor of EGFR and HER2, with IC_50_ values of 2 and 6 nM, respectively [470]. AEE788 also inhibits ABL1 (IC_50_ = 52 nM), FLT1 (IC_50_ = 59 nM), FMS (IC_50_ = 60 nM), SRC (IC_50_ = 61 nM), KDR (IC_50_ = 77 nM), and HER4 (IC_50_ = 160 nM). The AEE788 and rapamycin combination induced apoptosis in SUM149PT as well as HCC1143 cells [430]. In contrast, this combination did not have antiproliferative effects or induce apoptosis in normal mammary cells (MCF10A cells) and renal cells (RPTEC cells). This combination abolished p-mTOR (S2448), while no significant inhibition of p-mTOR was observed after single-agent treatment. P-AKT (S473) and p-ERK1 (T202/Y204) were inhibited by AEE788, and this inhibition was sustained by the combination. In addition, siRNA-based screening further revealed that RPS6KA3, RPS6KA6, RPS6KB1, and RPS6KL1 appear to be synthetic lethal targets for the AEE788 and rapalog combination treatment. An interesting point was that treatment with rapalogs induced the expression of both protein and mRNA of cyclin D1, while the addition of AEE788 abolished the rapalog-induced cyclin D1 expression in TNBC cells. Furthermore, the AEE788 and rapalog combination downregulated the expression of cyclin B1 and CDK4.

The susceptibility of TNBC cells to the EGFRi and mTORi combination has been shown to be correlated with the *PIK3CA* mutation status. The gefitinib and everolimus combination was effective in *PIK3CA*-mutatnt CAL-51 cells but not in wild-type HCC1937 cells [442]. CAL-51 cells contain a heterozygous E542K mutation in the *PIK3CA* gene with the *PTEN*-null background [442,471]. These results are consistent with the fact that the *PIK3CA* mutation amplifies AKT activation by *PTEN* loss in TNBC cells [19]. The combination reduced the levels of p-4E-BP1 (T37/46) and p-mTOR (S2448) in susceptible cells. No significant effects were observed on p-AKT (S473), whereas downregulation of p-ERK (T202/Y204) was observed. The combination further reduced the levels of cyclin B1 and cyclin E1 protein and mRNA, with a concordant increase in G_1_ cell cycle arrest and apoptosis. Gene expression profiling analysis identified upregulation of genes involved in DNA damage repair and cell cycle progression, such as checkpoint kinase 1 (*CHEK1*), *CHEK2*, cyclin A1 *(CCNA1*), *CCNB1*, and *CCNE1*.

Another study further supported the notion that the mTORC1 serves as a promising target for sensitizing TNBC cells to EGFRis. Combined treatment with gefitinib and MK-2206, a selective AKT inhibitor [420], synergistically reduced cell viability and colony formation of the TNBC cell lines HS578T and MDA-MB-213 [421]. More importantly, knockdown of the regulatory-associated protein of mTOR (RPTOR), but not rapamycin-insensitive companion of mTOR (RICTOR), confirmed the effects of MK-2206 in terms of the reductions in cell viability and total RPS6 levels in TNBC cells in the presence of gefitinib. Since RPTOR is the mTORC1-specific component [464], these results support the idea that the selective targeting of mTORC1 is a potential strategy to overcome EGFRi resistance.

Similar to small-molecule EGFR TKIs, a combinatorial benefit of vertical inhibition has also been reported with an anti-EGFR antibody in combination with either a PI3Ki or an AKTi. Duligotuzumab is a dual-acting human IgG1 monoclonal antibody against EGFR and HER3 [472,473]. Combined treatment with duligotuzumab and ipatasertib (a pan-AKTi) [384] or pictilisib (a pan-PI3Ki) [385] showed anticancer effects in TNBC cells both in vitro and in vivo, with prolonged inhibition of EGFR or HER3 signaling [159]. As expected, the duligotuzumab and pictilisib combination was more effective than the cetuximab and pictilisib combination in the regression of tumor growth of HCC70 xenografts, since inhibition of EGFR, AKT, or PI3K induced the abundance of HER3 [474,475,476], which was inhibited by duligotuzumab but not by cetuximab.

#### 4.1.4. Combination with RAS/RAF/MEK/ERK Inhibitors

Treatment with the MEK inhibitor selumetinib was shown to induce p-AKT (S473) in a panel of TNBC cells [393]. Combined treatment with selumetinib and gefitinib synergistically reduced the viability of TNBC cells and induced G_0_/G_1_ cell cycle arrest and apoptosis in selumetinib-sensitive SUM149PT cells. The gefitinib and selumetinib combination showed nearly complete inhibition of p-ERK1/2 (T202/Y204) and a further reduction in the selumetinib-mediated p-AKT (S473) level. In contrast, this combination did not induce G_0_/G_1_ cell cycle arrest and did not reach the IC_50_ value in the selumetinib-resistant TNBC cells MDA-MB-468 and SUM159PT. However, the molecular mechanism of this difference remains elusive.

#### 4.1.5. Combination with IKK/NF-κB Inhibitors

The therapeutic potential for targeting NF-κB in combination with EGFRis has been demonstrated by bortezomib [380]. Bortezomib (Velcade^®^) is the first FDA-approved proteasome inhibitor for the treatment of multiple myeloma [477]. One mode of action of bortezomib is downregulation of NF-κB activity through blocking proteasomal degradation of IκB [379,478]. As mentioned, activation of the NF-κB pathway has been described in ER-negative breast cancer cells [209,479]; however, a phase 2 clinical trial resulted in limited benefits [480]. Lapatinib, but not gefitinib or erlotinib, has been demonstrated to be able to induce activation of NF-κB through SRC-dependent p65 and IκBα phosphorylation [380]. Studies have shown that lapatinib upregulates SRC activity in an EGFR/HER2-independent manner. This lapatinib-induced NF-κB activation leads to the synergistic anticancer activity of co-treatment with lapatinib and bortezomib both in vitro and in vivo in two TNBC cells, HS578T and MDA-MB-231 [380]. Consistent with this finding, our group has identified that the combination of an IKK inhibitor and the EGFRi gefitinib has an anticancer effect in TNBC cells (You et al., manuscript in preparation).

#### 4.1.6. Combination with JNK Inhibitors

JNK-IN-8 is the first irreversible JNK inhibitor for JNK1, JNK2, and JNK3, with IC_50_ values of 4.7, 18.7, and 1 nM, respectively [408]. It covalently binds to C116 of JNK1 and JNK2, inhibiting phosphorylation of its target, c-Jun, at S63. Recently, the combination of lapatinib and JNK-IN-8 has been demonstrated to induce apoptosis of the TNBC cell lines, HCC1569, MDA-MB-231, and MDA-MB-435, in vitro and to retard MDA-MB-231 xenograft tumor growth in vivo [231]. The lapatinib and JNK-IN-8 combination reduced the transcriptional activities of NF-κB, activating protein 1 (AP-1), and NRF2 in TNBC cells, leading to an increase in the ROS level. As mentioned, NRF2 is a master regulator of a battery of genes involved in antioxidant responses, drug resistance, and detoxification [155]. Of importance, *NRF2*-KD reduces the expression of CSC markers, such as *ALDH1A1* and *ALDH3A1,* in pancreatic cancer cells [481].

#### 4.1.7. Combination with CDK Inhibitors

In humans, twenty-one cyclin-dependent kinases (CDKs) have been identified based on sequence similarity [482]. The CDK family is composed of two major subgroups of protein kinases: (1) those that regulate cell cycle progression (CDK1, CDK2, CDK4, and CDK6); (2) those that play roles in transcriptional processes (CDK7, CDK8, CDK9, CDK12, and CDK13) [483,484,485]. The functions of the remaining CDKs are still under investigation [482,484]. Dysregulation of CDKs has been associated with tumorigenesis or cancer progression, and they have been well established as anticancer targets [484,485].

Cell cycle progression is regulated by interplay between CDKs, cyclins, retinoblastoma (RB), CDK inhibitors, and transcription factor E2Fs [486,487]. Matrix metalloproteinase-17 (MMP17; also known as membrane-type-4 matrix metalloproteinase, MT4-MMP) has been identified as a copartner and is co-expressed with EGFR in approximately 80% of clinical TNBC samples. Its expression sensitizes TNBC cells to erlotinib [488,489]. In a subsequent study, dual targeting of EGFR and CDK4/6 by erlotinib and palbociclib, respectively, is additively effective in reducing tumor growth in TNBC xenografts and PDX cells expressing MMP17, EGFR, and RB, whereas PDX-TNBC cells without RB expression were resistant to this combination [428]. Interestingly, the expression of these markers has been found in approximately 50% of TNBC samples, which is useful to predict the sensitivity of TNBC to EGFR and CDK4/6 dual inhibition.

CDK9 is a catalytic subunit of a multiprotein complex, positive transcription elongation factor b (P-TEFb) [490], and is activated by CDK7 [491]. Both CDK9 and CDK7 positively activate gene transcription through sequential phosphorylation of the C-terminal domain of RNA polymerase 2 at S5/7 and S2, respectively [492,493,494].

CDK7 has been noted as a novel anticancer target, and its mRNA expression is linked with a poor prognosis in TNBC [495]. A covalent inhibitor of CDK7, THZ1 [437], has been reported to effectively repress the proliferation of breast cancer cells [496,497]. Very recently, the combination of THZ1 and erlotinib was identified to have synergistic or additive anticancer effects on various subtypes of breast cancer in vitro, including TNBC [438]. Although THZ1 represses a variety of genes such as *CDKN1B*, *MYC*, *EGFR*, *FOXC1*, *PLK2*, and *CTED2*, the mechanism of action of the combination of erlotinib and THZ1 remains to be elucidated.

A dual PKI of cell division cycle 7-related protein kinase (CDC7) and CDK9, PHA-767491, has been identified as a sensitizer of EGFRi in TNBC cells through a high-throughput screening of 273 PKIs in combination with lapatinib [99]. The synergism of the lapatinib and PHA-767491 combination in antiproliferative activity has been confirmed across 17 TNBC cell lines. This combination reduced CDC7 levels and phosphorylation of its downstream target, minichromosome maintenance protein 2 homology (MCM2), at S40/41. Since MCM2 is a crucial component of DNA helicase [498], the reduction of p-MCM2 suggests inhibition of DNA replication initiation by this combination. In addition, this combination reduced the CDK9-mediated phosphorylation of the DNA-directed RNA polymerase 2 subunit A (POLR2A) and the level of total POLR2A. This synergism is EGFR-specific, since EGFR TKIs, such as erlotinib and gefitinib, also resulted in synergism with PHA-767491. The lapatinib and PHA-767491 combination further repressed the levels of CDK4, cyclin D1, and p-RB (S780), with a concordant reduction in the G_0_/G_1_ phase and induction of G_2_/M cell cycle arrest. EGFRi and CDC7/CDK9i-induced G_2_/M cell cycle arrest is consistent with abnormal DNA replication origin activation checkpoint in cells, with loss-of-function *p53* mutations that are commonly found in TNBC cells [99,120,192,240,499]. This combination also induced apoptosis in TNBC cell lines. In clinical ER-negative breast cancer samples, high expression levels of CDC7 and POLR2A were associated with a poor prognosis [99].

#### 4.1.8. Combination with SFK Inhibitors

Human SRC family kinases (SFKs) are a group of non-receptor tyrosine kinases, including B lymphocyte kinase (BLK), Gardner–Rasheed feline sarcoma viral oncogene homolog (FGR), FYN-related kinase (FRK), proto-oncogene c-Fyn (FYN), hematopoietic cell kinase (HCK), lymphocyte-specific protein tyrosine kinase (LCK), Lck/Yes-related novel protein tyrosine kinase (LYN), v-src avian sarcoma (Schmidt-Ruppin A-2) viral oncogene homolog (SRC), and v-yes-1 Yamaguchi sarcoma viral oncogene homolog 1 (YES1) [500,501]. SFKs play crucial roles in regulating signal transduction provoked by cell surface receptors.

As mentioned earlier, anti-EGFR antibodies may exert an agonistic action on EGFR, leading to resistance of TNBC cells to anti-EGFR antibody therapeutics (see Section 3.2. Activation of Bypass Signaling Pathways). Recently, the combination of cetuximab and the SFK inhibitor PP2 was found to overcome this resistance in the TNBC cell lines MDA-MB-231 and MDA-MB-468 [142]. Cetuximab alone induced phosphorylation of PI3K and SRC. In addition, cetuximab activated RTKs, including IGF1R and VEGFR2. The combination of cetuximab and PP2 abolished cetuximab-induced p-IGF1R and p-VEGFR2, with a concordant reduction in cell proliferation.

Dasatinib is an orally available TKI of ABL1, SRC family kinases, KIT, and PDGFRα/β, and has been approved for the treatment of chronic myeloid leukemia (CML) and Philadelphia-chromosome-positive acute lymphoblastic leukemia (Ph+ ALL) (Table 4) [502,503]. Treatment with dasatinib alone reduced p-EGFR (Y845) and p-SRC (Y416), with a concordant reduction in nuclear EGFR and SRC and induction of radio-labeled cetuximab binding to the cell surface in TNBC cell lines such as MDA-MB-231 and MDA-MB-468 cells [440]. In an MDA-MB-468 xenograft model, the cetuximab and dasatinib combination further reduced the tumor volume compared with dasatinib alone, while no significant change was observed in an MDA-MB-231 xenograft model with this combination treatment [440]. Although the noted difference in these cell lines was a *KRAS* mutation (MDA-MB-468, wild-type vs. MDA-MB-231, mutated), further study is needed to determine the contributions of *KRAS* mutations to the differential effects of this combination.

A benefit of the combination of dasatinib with the EGFRi afatinib has also been reported [441]. The afatinib and dasatinib combination was synergistic or additive in 13 out of 14 TNBC cell lines. Interestingly, low levels of BCL2 and mTOR were associated with the synergism of this combination. P-EGFR (Y1068) and p-SRC (Y527) levels were commonly suppressed by the afatinib and dasatinib combination, with a concordant increase in G_1_ cell cycle arrest and without significant induction of apoptosis. The anticancer effect of this combination was confirmed in a TNBC xenograft model with decreases in CDC42 and p-EGFR (Y1068) in the xenograft tumor [441].

#### 4.1.9. Combination with ABL1 Inhibitors

Additional blocking of Abelson murine leukemia viral oncogene homolog 1 (ABL1) by imatinib overcomes EGFRi resistance in TNBC cells. Co-treatment of the TNBC cell lines HCC1806, MDA-MB-231, MDA-MB-468, and SUM159PT with imatinib and lapatinib resulted in a reduction in nuclear β-catenin accumulation, leading to the suppression of the tumor-promoting transcription factor MYC [397]. More interestingly, the expression of the long non-coding RNA (lncRNA) *HOX* antisense intergenic *RNA* (*HOTAIR*) was suppressed by the imatinib and lapatinib combination treatment. Nuclear β-catenin has been shown to bind the promoter region of *HOTAIR* according to a chromatin immunoprecipitation (ChIP) assay. *HOTAIR* is known to promote tumor progression and is associated with a poor prognosis [504,505,506]. The combination also reduced the tumor size of the MDA-MB-231 xenograft [397].

#### 4.1.10. Combination with Focal Adhesion Kinase (FAK) Inhibitors

FAK1 and PYK2 (or FAK2) are non-receptor tyrosine kinases controlling cell shape, adhesion, and motility [410]. High expression levels of FAK1 and activated PYK2 are associated with TNBC [507,508,509]. High expression levels of EGFR and PYK2 have been further associated with a poor prognosis among patients with TNBC [161]. In addition, IL-25 has been demonstrated to activate PYK2 and induce EGFRi resistance in TNBC cells [347] (See Section 3.4.10. Induction of Autophagy).

The combination of the FAK1 inhibitor (FAK1i) PF573228 [409] or the PYK2 inhibitor (PYK2i) PF431396 [411] with gefitinib or erlotinib synergistically reduced TNBC cell proliferation in vitro and tumor growth in vivo [161]. This synergism was also confirmed by shRNA-based knockdown of *FAK1* or *PYT2*. The FAK1i/PYK2i and EGFRi combination inhibited the activation of the AKT/S6K, STAT3, and ERK1/2 pathways (Table 3). Furthermore, PYK2 blocking reduced EGFRi-induced HER3 upregulation through N-myc downstream-regulated gene 1 (NDRG1)-mediated proteasomal degradation. Mechanistically, PYK2 blocking upregulated NDRG1 expression, leading to enhanced interactions of HER3 with its E3 ubiquitin ligase, NEDD4. NDRG1 is a metastasis suppressor in human cancers such as breast, colon, and prostate cancers [510]. Of importance, NRDG1 interacts with multiple signaling pathways, including the PI3K/AKT/mTORC1, RAS/RAF/MEK/ERK, and IKK/NF-κB pathways. However, the mechanism of EGFRi-induced NRDG1 expression remains to be determined. Co-targeting of PYK2 by PF431396 has also been reported to suppress the gefitinib-induced rebound of p-ERK1/2 (T202/Y204) and p-STAT3 (Y705) in MDA-MB-468 cells [424].

#### 4.1.11. Combination with Rho-Associated, Coiled-Coil-Containing Protein Kinase (ROCK) Inhibitors

Two in vivo and in vitro knockdown screenings have identified ROCK1 as a therapeutic target for TNBC [405]. Co-treatment with gefitinib and the ROCK inhibitor (ROCKi) GSK269962A reduced colony formation of a panel of TNBC cell lines, such as BT549, CAL-20, CAL-51, HCC38, HCC1806, HS578T, LM2, and MDA-MB-231 cells, compared with single-drug treatments. The combination inhibited the S phase and increased G_2_ cell cycle arrest. Cell cycle progression proteins, including cyclin A, CDK2, and p27, were consistently reduced, and the level of p-RB (S807/811) was also reduced in MDA-MB-231 cells treated with the combination, whereas little or no change in the levels of these proteins were observed in MDA-MB-231 cells treated with a single agent. In an orthotopic xenograft model with HCC1806 cells, tumor growth was reduced by the gefitinib and GSK269962A combination compared with the control or single agent treatment. Interestingly, the effect of the ROCKi alone in vivo was superior to that of gefitinib and not statistically different from that of the combination [405]. A subsequent study provided a more in-depth understanding of the mechanism of action of the gefitinib and GSK269962A combination [339]. Proteomic profiling of TNBC cell lines treated with the combination was compared to that of cells treated with a single drug alone; gefitinib was found to induce autophagy in TNBC cells, while the addition of the ROCKi blocked EGFRi-induced autophagy, leading to an anticancer effect in TNBC cells. In addition, the fact that another EGFRi afatinib [94,95] and ROCKi fasudil [511] resulted in similar anti-colony formation effects in TNBC cells suggests that the effect is a target-specific but not compound-specific [405]. Notably, fasudil has been approved in Japan and China, but not by the US FDA or the European Medicines Agency (EMA) [512].

#### 4.1.12. Triple Combination of PKIs

Triple combinations have been applied to suppress rewired signaling pathways by single or dual inhibition [22]. In cancer cells, blocking two pathways with a combination may induce rewiring of a signaling pathway(s) to circumvent the inhibition of survival signals [102,103]. Additional blocking of these rewired signaling pathways might provide another opportunity. For example, dasatinib attenuates SRC signaling induced by the poly(ADP-ribose)polymerase inhibitor (PARPi) veliparib and the DNA damaging agent carboplatin in TNBC [513]. Dasatinib is an ATP-competitive kinase inhibitor of ABL1, SRC, KIT, and CSK [374,375,502]. SRC is a member of the SFKs, and its overexpression has been associated with aggressiveness of tumors, including TNBC [514,515,516,517]. SFKs are key components in cell signaling regulation (see Section 4.1.8. Combination with SFK Inhibitors).

An earlier study demonstrated that a triple combination of cetuximab, dasatinib, and cisplatin induced apoptosis synergistically in various TNBC cell lines [376]. In addition, the triple combination attenuated the cell growth and migration of TNBC cells. Single (cetuximab or cisplatin) or dual (cetuximab + cisplatin) treatment induced p-EGFR, p-AKT, and p-ERK in a TNBC cell line-dependent manner, while the addition of dasatinib attenuated this activations [376]. Dasatinib may further contribute to the inhibition of EGFR through blocking of SRC-induced EGFR phosphorylation [376] and direct EGFR binding [518], and by inducing its lysomomal degradation [519]. Interestingly, dasatinib has been shown to reduce the expression of *ALDH1A1*, leading to potentiation of gemcitabine in a gemcitabine-resistant pancreatic cancer cell line [520]. The ALDH family members consist of 19 isoforms, which have been proposed as hallmarks of drug resistance in CSCs [521]. The expression of *ALDH1A1* and *ALDH3A1* has also been linked to NRF2 in pancreatic cancer cells. *NRF2*-KD abolished the expression of *ALDH1A1* and *ALDH3A1*, leading to sensitization of pancreatic cancer cells to 5-fluorouracil (5-FU) [480].

A combination screening of 33 FDA-approved PKIs identified a triple combination of afatinib, dasatinib, and trametinib as an effective strategy for TNBC treatment [444]. The drug combination discovery approach was designed to use a single-mixture solution of 33 PKIs at the start and then to use dropout solutions in the TNBC cell line HCC1143. The dropout solutions were made by exclusion of a group of kinase inhibitors that were classified according to their primary targets from the 33X mixture solution. The triple combination displayed antiproliferative activity in two TNBC cell lines, HCC1143 and MDA-MB-468. Although the mechanism of action remains to be investigated, the results suggest that simultaneous inhibition of the PI3K/AKT and MEK/ERK pathways is a promising approach to potentiate EGFRis in TNBC [444].

Very recently, the combination of gefitinib with the AKT inhibitor, AT7867, has been shown to induce activation of the MEK/ERK pathway in TNBC cell lines [22]. The addition of the MEK inhibitor PD-0325901 to the gefitinib and AT7867 combination resulted in the synergistic reduction of cell proliferation and colony formation of HS578T and MDA-MB-231 cells. Apoptotic cell death, evident with the increased subG_1_ phase, was only observed with the triple combination, with a concordant reduction in XIAP and increase in cleaved caspase-3. Interestingly, a reduction in the total RPS6 was observed in TNBC cells treated with the triple combination [22]. The importance of the total RPS6 in TNBC cell proliferation was previously demonstrated by *RPS6*-KD [15] (see Section 4.1.2. Combination with MET Inhibitors (METis)); thus, it is important to investigate the mechanisms of action of both intrinsic and acquired resistance to enrich our strategies to fight cancers.

**Figure 2:**
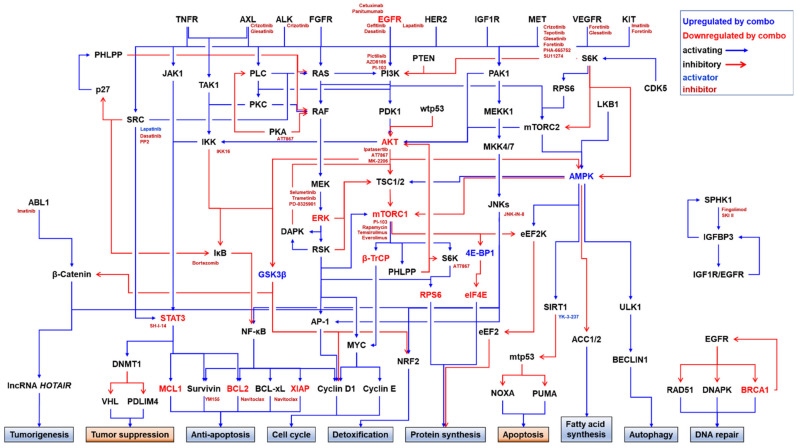
Schematic diagram of the putative EGFR and related signaling pathways in TNBC cells. Abbreviations: ABL1, Abelson murine leukemia viral oncogene homolog 1; ACC1/2, acetyl-CoA carboxylase 1/2; AKT, v-akt oncogene homolog; ALK, anaplastic lymphoma kinase; AMPK, 5′ adenosine monophosphate (AMP)-activated protein kinase; AP-1 activator protein 1; AXL, Anexelekto receptor tyrosine kinase; BCL2, B-cell lymphoma 2; BCL-xL, B-cell lymphoma-extra-large; BRCA1, breast cancer type 1 susceptibility protein; β-TrCP, beta-transducin repeat-containing protein; CDK5, cyclin-dependent-like kinase 5; MYC, cellular myelocytomatosis; DAPK, death-associated protein kinase; DNAPK, DNA-dependent protein kinase; DNMT1, DNA (cytosine-5)-methyltransferase 1; eEF2, eukaryotic elongation factor 2; eEF2K, eukaryotic elongation factor 2 kinase; EGFR, epidermal growth factor receptor; eIF4E, eukaryotic translation initiation factor 4E; ERK, extracellular-signal-regulated kinase; FBW7, F-box and WD repeat domain-containing 7; FGFR, fibroblast growth factor receptor; GSK3β, glycogen synthase kinase-3 beta; HER2, human epidermal growth factor receptor 2; IGF1R, insulin-like growth factor 1 receptor; IκB, nuclear factor of kappa light polypeptide gene enhance in B-cells inhibitor; IKK, IκB kinase; JAK1, Janus kinase 1; JNKs, c-Jun N-terminal kinases; KIT, v-kit Hardy-Zuckerman 4 feline sarcoma viral oncogene homology; LKB1, liver kinase B1; MCL1, myeloid-cell leukemia 1; MEK, MAPK/ERK kinase; MEKK1, mitogen-activated protein kinase kinase kinase 1; MET, mesenchymal–epithelial transition factor; MKK4/7, mitogen-activated protein kinase kinase 4; mTORC1/2, mammalian target of rapamycin complex 1/2; mtp53, mutant p53; NF-κB, nuclear factor of kappa light polypeptide gene enhanced in B-cells; PAK1, p21-activated kinase 1; PDLIM4, PDZ and LI domain 4; PHLPP, PH domain and leucine-rich repeat protein phosphatase; PI3K, phosphoinositide 3-kinase; PDK1, phosphoinositide-dependent kinase-1; PLC, phospholipase C; PKA, protein kinase A; PKC, protein kinase C; PTEN, phosphatase and tensin homolog; PUMA, p53-upregulated modulator of apoptosis; RAD51, RAD51 (*S. cerevisiae*) homolog; RAF, rapidly accelerated fibrosarcoma kinase; RAS, rat sarcoma; RPS6, ribosomal protein S6; RSK, ribosomal S6 kinase; S6K, S6 kinase; SIRT1, NAD-dependent deacetylase sirtuin-1; SRC, v-src avian sarcoma (Schmidt–Ruppin A2) viral oncogene homolog; STAT3, signal transducer and activator of transcription 3; TAK1, transforming growth factor beta-activated kinase 1; TNFR, tumor necrosis factor receptor; TSC1/2, tuberous sclerosis complex 1/2; ULK1, Unc-51-like autophagy-activating kinase 1; VEGFR, vascular endothelial growth factor receptor; VHL, Von Hippel–Lindau tumor suppressor; XIAP, X-linked inhibitor of apoptosis.

Cumulative evidence supports the potential for enhancing EGFRi-mediated anticancer efficacy in TNBC by using combination strategies targeting EGFR and related signaling pathways in TNBC cells (Figure 2). As mentioned earlier, crosstalk and rewiring of signal transduction pathways contribute to either intrinsic or acquired EGFRi resistance in TNBC. Continuous efforts will provide a comprehensive understanding of the molecular mechanisms of EGFRi resistance and rationale for circumventing the limited efficacy of EGFRi in TNBC. The availability of small-molecule inhibitors for enzymes other than protein kinases will also facilitate future studies (see the following sections).

### 4.2. Combination with Other Targeted Therapeutics

#### 4.2.1. Combination with PARP Inhibitors (PARPis)

PARP1 is the first therapeutic target of FDA-approved small-molecule inhibitors for TNBC treatment. Olaparib (Lynparza^®^) and talazoparib (Talzenna^®^) have been approved for germline *BRCA*-mutated metastatic breast cancer and germline *BRCA*-mutated, HER2-negative, locally advanced or metastatic breast cancer, respectively; however, their efficacy is extending beyond tumors based on *BRCA* mutation status [522,523].

An earlier study demonstrated that the combination of lapatinib and the PARPi veliparib resulted in synergistic anti-clonogenic activity in the TNBC cell lines MDA-MB-231, MDA-MB-453, and MDA-MB-468 [372]. Of interest, these TNBC cell lines lack *BRCA1* mutations [524]. This combination also induced intrinsic apoptosis, as evidenced by activation of caspase-3 and caspase-9. This synthetic lethal interaction between lapatinib and veliparib is due to lapatinib-mediated homologous recombination repair deficiency, which mimics the *BRCA1* mutation. Furthermore, lapatinib induced the translocation of BRCA1 and EGFR to the cytoplasm, preventing DNA repair in the nucleus, with a consistent increase in the DNA damage marker γ-H2AX. In addition, lapatinib disrupted the BRCA1-EGFR interaction. This combination attenuated MDA-MB-231 xenograft tumors [372]. Recently, negative regulation of EGFR expression by BRCA1 through transcriptional activation of miR-146a that targets the 3′-untranslated region (3′-UTR) of EGFR has been reported [525]. Mutations of *BRCA1* have been linked to the TNBC phenotype [19,20].

Very recently, PARPi-resistant, *BRCA1*-mutated SUM149PT TNBC cell lines were developed, which demonstrate high levels of p-MET (Y1234/1235) and p-EGFR (Y1086) [436]. Knockdown of *MET* reversed PARPi resistance, while the triple combination of an EGFRi (gefitinib), a METi (crizotinib), and a PARPi (talazoparib) further reduced cell viability [436]. BEZ235, an inhibitor of PI3K, mTOR, ATM, ATR, and DNA-PK, has been identified as a potentiator of the PARPi olaparib in reducing colony formation of two *BRCA1*-mutated TNBC cell lines, MDA-MB-436 and SUM149PT, with reductions in olaparib-induced 53BP1 foci in SUM149PT cells [526]. Further studies on the interactions between EGFRis and PARPis will extend our understanding of TNBC biology and therapy.

#### 4.2.2. Combination with Inhibitors of Anti-Apoptotic Proteins

ABT-806 is a humanized, tumor-specific anti-EGFR IgG1 monoclonal antibody [527]. The antibody–drug conjugates (ADCs) ABT-414 (depatuxizumab mafodotin) [528] and ABBV-321 (serclutamab talirine) [529] are composed of the antibody ABT-806 conjugated to the cytotoxic monomethyl auristatin F (MMAF) and the affinity-maturated version of ABT-806 conjugated to an ultrapotent pyrrolobenzodiazepine dimer, respectively. Navitoclax (ABT-263) is a small-molecule inhibitor of BCL-xL, BCL2, and BCL2L2 with anticancer activity [433]. The combination of navitoclax with these ADCs has been identified as an effective strategy in multiple PDX models of TNBC [258]. After fourteen days of daily navitoclax treatment with ABT-414 once per week, five of seven PDX models showed reduced tumor growth and three of seven models showed tumor regression compared with the vehicle control. A further study involving daily navitoclax treatment for 5 days per week and MMAF-loaded ADC (ABT-414 or AB095-MMAF, a non-tumor-targeted ADC) treatment once per week including single-agent controls was performed. Interestingly, treatment with ABT-414 alone showed no effect, while treatment with navitoclax alone reduced the tumor volume by approximately 20%. The ABT-414 and navitoclax treatment further induced tumor regression by approximately 40% [258]. The combination effect was EGFR-mediated, since the non-tumor (tetanus toxoid)-targeted ADC had little or no synergistic effect with navitoclax. The combination effect of an alternative ADC, ABBV-321, with navitoclax further supports EGFR dependency.

A screening of 1363 drugs in vitro in ten patient-derived TNBC cell lines identified that the combination of afatinib and YM155, an inhibitor of baculoviral IAP repeat-containing protein 5 (*BIRC5*; gene encoding survivin) expression [448], reduced the growth of patient-derived TNBC cells in vitro and the PDX mammary tumors in vivo [449]. However, the effect of the combination did not reach statistical significance compared with treatment with YM155 alone. This dominant effect of YM155 might be due to its ability to reduce EGFR expression in TNBC cells through an unknown mechanism. Clinically, high expression levels of EGFR and survivin are associated with metastasis-free survival [449].

As mentioned earlier (see Section 3.4.2. Overexpression of Anti-apoptotic Proteins), MCL1 has been reported to confer EGFRi resistance in an ELP complex-dependent manner [260]. In addition, co-treatment of the MCL1 inhibitor S63845 synergistically induced erlotinib sensitivity in TNBC cells. Further studies on the underlying mechanism of ELP-complex-dependent MCL1 expression will be of interest in understanding the newly identified oncogene addition in TNBC [260].

#### 4.2.3. Combination with Sphingosine Kinase (SPHK) Inhibitors

SPHK1 has been linked to acquired drug resistance of various cancers to chemotherapeutics and receptor inhibitors [530,531,532,533,534,535,536,537,538]. A high level of SPHK1 has also been associated with poor OS and PFS among patients with TNBC [539]. One study demonstrated that the combination of the SPHK inhibitor SKI II with gefitinib reduced the growth of MDA-MB-468 xenograft tumors [389]. SPHK1 converts sphingosine to sphingosine 1-phosphate (S1P), which provokes a variety of signaling pathways via binding to S1P receptors 1–5 (S1PR1-5). SPHK1 has been established as an oncogene, and its overexpression is associated with a poor clinical prognosis in most cancers [538,540]. More interestingly, a higher expression of SPHK1 has been found in TNBC than in ER-positive breast cancer and is associated with a poorer DFS [541]. In TNBC cells, pharmacological inhibition or siRNA-based knockdown of SPHK1 inhibits insulin-like growth factor-binding protein-3 (IGFBP3)-enhanced EGFR activation. Although the underlying molecular mechanism is still largely unknown, the combination of an SPHK1 inhibitor and gefitinib significantly reduced tumor growth in an MDA-MB-468 xenograft model [389].

Another SPHK1 pathway inhibitor, fingolimod (FTY720) [412,542], which is a clinically approved S1PR1 antagonist [543], has also been demonstrated as a potentiator of gefitinib in TNBC cells, especially in BL subtype cells, in vitro and in vivo [413]. The gefitinib and fingolimod combination is also antiproliferative in HCC70 (a BL2 subtype), MDA-MB-231 (an MSL subtype), and BT549 (an M subtype) cells. The shRNA-based knockdown of *IGFBP3* abolished the synergism induced by the gefitinib and fingolimod combination. CD44 has been reported to be upregulated by both EGFR [544] and SPHK1 [545] and to be associated with a poor prognosis of TNBC [546] (see Section 3.4.8 for CD44 in EGFRi resistance). In addition to CD44 downregulation by gefitinib alone, further downregulation occurs with the addition of fingolimod to gefitinib in TNBC cell lines [413]. Furthermore, the gefitinib and fingolimod combination extended the survival of TNBC xenograft nude and syngeneic immune-competent mice. The DNA-damaging agent induced direct binding and nuclear co-localization of IGFBP3 to EGFR in TNBC cells [547]. In HCC1806 and MDA-MB-468 xenograft tumors, nuclear IGFBP3 was reduced in response to each drug and further reduced by the combination [413]. However, the mechanism of action of this combination remains to be investigated. Notably, a recent study demonstrated that the inhibition of SPHK1 activity either by siRNA or by PF-543, a sphingosine-competitive SPHK1 inhibitor [548], led to suppression of the Notch pathway, with reductions in migration and invasion of TNBC cells in vitro and in vivo [539]. In addition, blocking SPHK1 activity also enhanced the sensitivity of TNBC cells to 5-FU and doxorubicin.

#### 4.2.4. Combination with ARF Exchange Activity Inhibitors (ARFis)

The ARFi brefeldin A (BFA) [549] has been demonstrated to sensitize MDA-MB-231 cells to gefitinib, leading to a synergistic increase of apoptosis [288]. In addition, BFA potentiates the PI3K inhibitor LY294002 and the SFK inhibitor PP2 but not the MEKi PD0325901. BFA increases cytotoxicity induced by the gefitinib and PP2 combination but not the gefitinib and LY294002 combination or the gefitinib and PD0325901 combination. The gefitinib + BFA + PP2 combination further reduced the levels of p-AKT, p-ERK1/2, and p-SRC in MDA-MB-231 cells. Results from siRNA-based knockdown and overexpression of ARF1 further support that the targeting ARF1 synergistically induced the gefitinib-mediated apoptosis in MDA-MB-231 cells [288].

#### 4.2.5. Combination with Protein–Protein Interaction Inhibitors (PPIis)

Targeting of the protein–protein interaction (PPI) to control disease has been the most challenging task. However, recent advances have resulted in approval of PPIis for marketing [550]. Disrupting the BAG3-HSP70 interaction has been demonstrated to sensitize TNBC cells to EGFRis [419]. BCL2-associated athanogene 3 (BAG3) is a co-chaperone for heat shock protein 70 (HSP70) and heat shock cognate 71 kDa (HSC70) chaperone proteins [551,552]. High expression levels of BAG3 mRNA and protein have been identified in approximately 50% of TNBC cell lines and clinical samples, and high mRNA levels are correlated with a poor DFS [419]. A small-molecule inhibitor of the BAG3-HSP70 interaction, YM-1 [418], enhanced the cetuximab-induced reduction of the cell viability in two TNBC cell lines, BT549 and MDA-MB-468 [419]. Similarly, knockdown of *BAG3* also sensitized these cells to cetuximab. Both YM-1 and *BAG3*-KD targeted p-AKT (S473) and p-FAK (Y397). *BAG3*-KD also reduced TNBC cell proliferation, migration, and invasion in vitro [419]. These results further suggest the importance of the PI3K/AKT and FAK pathways in regulating EGFRi resistance and a novel alternative strategy to overcome this issue. Exploring novel PPIis may expand the potential therapeutic options for TNBC treatment.

### 4.3. Combination with Conventional Chemotherapeutics

Conventional chemotherapeutics induce non-selective tumor cell killing and include microtubule stabilizers (vinblastine, paclitaxel, and docetaxel), DNA alkylators (cisplatin and carboplatin), anthracyclines, nucleoside analogues and nuclobases, topoisomerase inhibitors (camptothecin), and metabolic inhibitors (5-FU and methotrexate) [553].

#### 4.3.1. Combination with Microtubule Stabilizers

Epothilones, which are similar to taxanes, stabilize tubulin to prevent cancer cell division [554]. Ixabepilone, a semisynthetic analog of epothiolone B [555], has been combined with gefitinib to treat TNBC cells both in vivo and in vitro. The gefitinib and ixabepilone combination reduced the growth of SUM159PT xenograft tumors compared with single treatment with both drugs [406]. Although statistical significance was not achieved, the combination also further reduced the growth of MDA-MB-231 tumors. In vitro, the gefitinib and ixabepilone combination reduced the autophagy markers p62 and LC3B (microtubule-associated proteins 1A/1B light chain 3B) in both TNBC cells. The effect of this combination on the mammosphere formation efficiency, both in vitro and in vivo, also did not reach statistical significance. To date, the clinical results of the combination of EGFRis and ixabepilone have shown limited efficacy (see Section 5. Clinical Studies).

#### 4.3.2. Combination with DNA-Damaging Agents

DNA-damaging agents include a broad range of compounds that demonstrate anticancer activity through either covalent or non-covalent DNA binding [556].

Cisplatin (cis-diamminedichloroplatinum 2, CDDP) interacts with DNA and forms DNA adducts [557]. Cisplatin inhibits the proliferation of various cancer cells and activates DNA damage responses. The combination of cisplatin with cetuximab induced apoptotic cell death and antiproliferative effects in MDA-MB-468 cells, while cetuximab alone failed to induce apoptotic cell death [365]. This synergism demonstrated no dose response of cetuximab. In addition, the cetuximab and cisplatin treatment induced an increase in BRCA1 and a decrease in EGFR [365]. Consistent with this finding, BRCA1 has been demonstrated to downregulate *EGFR* by targeting its 3′-UTR by miR-146a [525]. BRCA1 transactivates miR-146a by binding to its promoter.

Anthracyclines, such as daunorubicin, doxorubicin, epirubicin, and idarubicin, are chemotherapeutics extracted from *Streptomyces* species [558]. The combination of cetuximab or panitumumab with cisplatin or epirubicin showed synergistic effect in decreasing cell proliferation of the *BRCA1*-mutated TNBC cell line SUM1315MO2, which has a wild-type *KRAS* and *PTEN* background, whereas no effect was found in another *BRCA1*-mutated TNBC cell line, HCC9137, with *PTEN* deficiency [407]. The TNBC cell lines with wt*BRCA1*, MDA-MB-231 (*KRAS*-mutated), and MDA-MB-468 (*PTEN*-null) also were not affected by this combination. Of interest, restoration of wtBRCA1 abolished this synergism. Anti-EGFR antibodies as a monotherapy did not affect the cell cycle distribution but DNA-damaging agents alone induced marked G_2_ cell cycle arrest. The combination of anti-EGFR antibodies and DNA-damaging agents redistributes the cell cycle by reducing G_2_ and increasing G_1_ cell cycle arrest. No additional increase in DNA-damaging, agent-induced apoptosis was observed [407]. One plausible explanation for the differential effects of the combination found in SUM1315MO2 and HCC1937 cells is activation of the PI3K/AKT pathway, since *PTEN*-deficiency may cause activation of PI3K/AKT signaling in HCC1937 cells [559]. Further studies are needed to elucidate the mechanism of action for this combination.

#### 4.3.3. Combination with Nucleoside Analogues and Nucleobases

Cytotoxic nucleoside analogues and nucleobases are antimetabolites that inhibit DNA or RNA synthesis through incorporation into growing DNA or RNA strands and termination of their extension [560]. These agents are used as antiviral or anticancer chemotherapeutics. These drugs include (1) the purine analogues fludarabine and cladribine, (2) the pyrimidine analogues cytarabine and gemcitabine; and (3) the fluoropyrimidines fluorouracil and capecitabine [560].

Gemcitabine is a cytidine analogue that is widely used to treat cancers such as bladder cancer [561], breast cancer [562], NSCLC [563], ovarian cancer [564,565], and metastatic pancreatic cancer [566] as a single agent or in combination with other chemotherapeutics. After entering cells, gemcitabine is converted into various nucleotides, including difluorodeoxycytidine triphosphate (dFdCTP), difluorodeoxycytidine diphosphate (dFdCDP), and difluorodeoxyuridine monophosphate (dFdUMP) [567]. These metabolites inhibit DNA polymerase (dFdCTP) [568], ribonucleotide reductase (dFdCDP) [569,570], and thymidylate synthase (dFdUMP) [571].

Gemcitabine has been reported to overcome erlotinib resistance in erlotinib-resistant, EGFR-overexpressing A431 cells by downregulating p-AKT levels [11]. Similar to this squamous carcinoma cell line, gemcitabine also reduced p-AKT in the TNBC cell lines BT549 and MDA-MB-468 and synergistically reduced the viability of TNBC cells in combination with erlotinib [11].

#### 4.3.4. Combination with Multiple Chemotherapeutics

Similar to PKIs, the anticancer effects of EGFRis in combination with multiple chemotherapeutics have been investigated. A triple combination of gefitinib, carboplatin, and docetaxel showed synergism in antiproliferation assays [366]. This triple combination was superior to gefitinib and carboplatin or gefitinib and docetaxel in antiproliferation assays. Furthermore, this triple combination induced a similar level of G_2_/M cell cycle arrest compared with the combination of carboplatin and docetaxel [366].

### 4.4. Other Combination Strategies

#### 4.4.1. Combination of Anti-EGFR Antibodies

A strategy using combinations of non-competitive anti-EGFR antibodies has been suggested to achieve robust degradation of EGFR, leading to tumor inhibition [381]. Administration of a mixture of monoclonal antibodies (mAbs) may have resulted in synergistic effects because individual mAbs have partial antitumor effects based on diverse neutralizing effects, such as inhibition of ligand binding, prevention of receptor dimerization, and induction of receptor internalization. The combination of panitumumab and mAb111 reduced surface EGFR proteins by enhancing their internalization and degradation. Diminished surface EGFR led to reduction of TNBC cell invasion and colony formation in vitro and tumor growth in vivo [381].

A mixture of six monoclonal antibodies against EGFR, HER2, and HER3 was reported to reduce the growth of fifteen TNBC PDX tumors in vivo [446]. Treatment with this Pan–HER antibody mixture reduced the levels of p-EGFR (Y1068) and p-HER3 (Y1289) and their total protein levels, with consistent reductions in their downstream effectors p-AKT (T308), p-ERK1/2, and p-FAK (Y397) in PDX tumors in vivo. The RT-PCR-based expression analysis of 88 EGFR-associated genes demonstrated the downregulation of genes such as *RAS*, *RAF*, *MEK*, *ERK*, *JNK*, *c-Jun*, *c-Fox*, *JAK*, *STAT3*, *IKK*, *NF-κB p52*, and *p65/RelA* [446]. These results are consistent with the EGFR-NF-κB crosstalk previously reported (see Section 3.3.3. Activation of the NF-κB Pathway).

#### 4.4.2. Combination in a Single Molecule: Bispecific Antibody

A single-chain diabody is a recombinant multivalent and bispecific single-chain antibody fragment [572]. Recently, a tetravalent, bispecific single-chain diabody-Fc fusion protein targeting EGFR and HER3 was developed, and its anticancer efficacy in TNBC cells both in vitro and in vivo was demonstrated [445]. Since the kinase activity of HER3 is very low (see Section 3.2. Activation of Bypass Signaling Pathways), functional blocking of HER3 is achievable through antibodies binding to its ectodomain, but not with small-molecule TKIs [445]. The diabody, composed of the antigen-binding sites of a humanized cetuximab (IgG hu225) and IgG 3-43, which target EGFR and HER3, respectively, has been reported to downregulate p-EGFR (Y1068) and p-HER3 (Y1289), as well as their downstream effectors p-AKT (T308) and p-ERK (T202/Y204), in FaDu, a hypopharyngeal carcinoma cell line. The diabody was further demonstrated to reduce TNBC CSC (ALDH^High^) populations both in vitro and in vivo and consistently reduced TNBC tumor regrowth in vivo more efficiently than the parental antibodies alone or a combination of them [445].

#### 4.4.3. Combination with Autophagy Inhibitors

As mentioned earlier, autophagy has been established as a novel mechanism and therapeutic target to overcome anticancer drug resistance [331,332,333]. EGFR TKIs have been reported to activate autophagy as a cytoprotective response in various cancer cell lines, including breast cancer [333], lung cancer [573,574], squamous cell carcinoma, and transitional cell carcinoma [337]. Gefitinib-induced autophagy in two TNBC cell lines, MDA-MB-231 and MDA-MB-468, is inhibited by either 3-methyladenine (3-MA) or bafilomycin A1 (Baf-A1) [415]. Blocking the gefitinib-induced autophagy resulted in synergistic augmentation of cytotoxicity in TNBC cells in vitro and anticancer effects on TNBC tumors in vivo. The combination of gefitinib with autophagy inhibitors induced G_0_/G_1_ cell cycle arrest and accumulation of DNA damages as demonstrated by increases in the levels of phosphorylation of ataxia telangiectasia mutated (ATM), CHEK1, CHEK2, and γ-H2AX, a hallmark of DNA double-strand breaks [575]. The combination further induced apoptosis with induction of BCL2-associated X (BAX), cleaved caspase-3, and cytochrome C release, and a reduction in the anti-apoptotic protein BCL2 [415].

The combination of osimertinib and chloroquine (CHQ), an autophagy and lysosome inhibitor [576], has been reported to show anticancer effects, namely a reduction in cell viability in MDA-MB-231 cells [426]. Concordantly, LC3B-II, an autophagic marker that is tightly associated with autophagosomal membranes [577], was upregulated by this combination. Furthermore, the proapoptotic proteins, BCL2-associated agonist of cell death (BAD) [578] and active caspase-3, were increased by this combination. These results suggest that the osimertinib and CHQ combination exerts anticancer effects through autophagy–apoptosis crosstalk, but further details remain to be determined [426].

Interestingly, a recent study demonstrated that blocking autophagosome clearance with the combination of gefitinib and ROCKi (GSK269962A) might induce antitumor activity in TNBC [339] (see Section 4.1.11. Combination with Rho-Associated, Coiled-Coil-Containing Protein Kinase (ROCK) Inhibitors).

#### 4.4.4. Combination with Antioxidants

Inhibiting ROS is a potential approach to overcome EGFRi resistance. Previously, co-treatment of genetically engineered catalase sensitized the TNBC cell line MDA-MB-468 to gefitinib [373,417]. In MDA-MB-468 cells, a high level of ROS induced tyrosine phosphorylation of EGFR [373]. Transduction of genetically engineered catalase (CAT-SKL) reduced ROS-mediated EGFR phosphorylation and enhanced gefitinib cytotoxicity in two TNBC cell lines, MDA-MB-468 and SUM149PT [373]. A subsequent study demonstrated that CAT-SKL inhibits CSCs and erlotinib inhibits non-CSCs in a subset of TNBC cell lines, such as HCC70 and MDA-MB-468 cells [417]. CAT-SKL reduced the expression of methyl-CpG binding domain 2 isoform c (MBD2c), leading to a reduction of mammosphere formation. Overexpression of MBD2c in MDA-MB-468 cells increased the number and size of mammospheres. Co-treatment with the antioxidant (−) epicatechin with erlotinib confirmed the use of the CAT-SKL and erlotinib combination [417]. These results imply the importance of targeting subpopulations of heterogenous tumor cells. Further studies on the role of MBD2c in CSCs may provide new insights for TNBC therapeutics.

#### 4.4.5. Combination with Natural Products

Icaritin is a natural compound from the *Epimedium* genus with anticancer activity [579]. The combination of cetuximab with icaritin reduced cell viability and induced apoptosis in the TNBC cell lines MDA-MB-231 and MDA-MB-436 [450]. This combination further resulted in a reduction of MDA-MB-231 and MDA-MB-436 xenograft tumor growth in vivo. Icaritin has been demonstrated to decrease both *EGFR* and *ERα36* expression in TNBC cells [580]. The expression of *ERα36* is associated with stemness and metastasis of breast cancer and chemoresistance in ER-negative breast cancer (see Section 3.4.5. Overexpression of the Estrogen Receptor Alpha (Erα) Variant).

#### 4.4.6. Combination with Gene Therapy

As mentioned earlier, *p53* mutations are commonly found in TNBC [120,192]. Since *p53* mutations result in gain-of-function as an oncogene as well as loss-of-function in terms of tumor suppressor function, targeting mtp53 or restoring wild-type p53 (wtp53) is a promising therapeutic option [239]. Notably, adenoviral-vector-expressing wtp53 (Ad-wtp53) was approved as the world’s first commercial gene therapy product by the Chinese regulatory authority [581]. Ad-wtp53 decreased the growth of MDA-MB-468 xenograft tumors in combination with gefitinib [398]. Combined treatment with Ad-wtp53 and gefitinib induced G_2_/M cell cycle arrest and apoptosis and reduced activation of the PI3K/AKT/mTORC1 pathway. It would be an interesting research topic to evaluate whether small-molecule inhibitors of mtp53, such as the SIRT1 activator YK-3-237 [240], augment EGFRi effects in TNBC cells.

The combination of cetuximab with an miR-155-5p antagomir has been demonstrated to enhance the anticancer effects of cetuximab in MDA-MB-468 cells through promoting apoptosis and pyroptosis, both in vitro and in vivo [453]. Pyroptosis is caspase-1-dependent programmed cell death and is proinflammatory in nature [582]. The common features of this combination effect in vitro and in vivo are the downregulation of p-EGFR and upregulation of gasdermin-E (GSDME). MiR-155-5p directly targets the 3′-UTR of *GSDME* mRNA [453]. Of importance, GSDME is a precursor protein of the pore-forming N-terminus of GSDME (GSDME-N) that converts apoptosis to pyroptosis [583,584]. Upon cleavage by caspase-3, GSDME-N binds to and perforates the plasma membrane to trigger inflammatory pyroptosis [584]. These results suggest the potential importance of pyroptosis as a novel mechanism of EGFRi potentiation by drug combination.

### 4.5. Immuno-Oncological Approaches

Since TNBCs have been identified as an immunogenic malignance [585,586,587,588], immuno-oncological approaches may be promising alternatives for TNBC treatment. The immuno-oncological approaches described in the following sections have not been fully explored yet in combination with EGFRis; the future studies on EGFR targeting in TNBC will be expected to include these new modalities.

#### 4.5.1. ADCC

Although cetuximab alone did not induce antiproliferative effects or apoptosis in TNBC cells in vitro, it did induce the NK-cell-dependent ADCC in vitro in EGFR-expressing TNBC cells [369]. This ADCC was further enhanced in vitro, ex vivo, and in vivo by interleukin (IL)-2 or IL-15, which stimulates NK cells to produce interferon (IFN)-γ [369,370]. The ADCC induced by the cetuximab and IL-2 or IL-15 combination was more evident, since the combination reduced the xenograft tumor volume with an increase in infiltrating NK cells, whereas no significant induction of direct antiproliferative effects on the tumor was observed [370]. A subsequent study reported that the combination of cetuximab and IL-15 further activated tumor killing by NK cells and stimulated the maturation of dendritic cells (DCs) in a co-culture experiment with the TNBC cell line IIB-BR-G [439]. Taken together, the combination of anti-EGFR antibodies and interleukin may enhance ADCC in the tumor microenvironment, even in the absence of a direct antitumor effect by anti-EGFR antibodies.

Recently, sacituzumab govitecan (Trodelvy^®^) received accelerated FDA approval for the treatment of patients with metastatic TNBC [589]. Sacituzumab govitecan is an antibody drug conjugate (ADC) composed of a humanized monoclonal antibody for trophoblast cell surface antigen 2 (Trop-2) and topoisomerase inhibitor SN-38 [590]. Sacituzumab govitecan was granted regular approval by the US FDA for TNBC on 7 April 2021 [591]. As for IL-2 and IL-15, it is of interest whether the combination of EGFRis and sacituzumab govitecan is effective for TNBC or not.

#### 4.5.2. Chimeric Antigen Receptor (CAR)-Engineered Cell Therapies

CAR-engineered immune cell therapy has been established as a promising immuno-oncologic strategy for cancer therapy. After the great success of CAR-engineered T (CAR-T) cell therapy [592], CAR-engineered NK (CAR-NK) cells [593] and CAR-engineered macrophages (CAR-M) [594] have been developed to reinforce the treatment of multiple cancers. The major challenge of CAT-T is that the clinical benefit is limited in hematologic cancers but not in solid tumors [595,596].

Cell surface antigens, including RTKs (e.g., AXL, EGFR, MET, ROR, etc.), which are overexpressed in TNBCs, are promising targets for CAR-T cell therapy [597]. Since in vitro and in vivo studies have demonstrated the efficacy of CAR-T cell therapy against TNBC cells, combination approaches may further expand the therapeutic opportunities in the future. In fact, CD32A^131R^–chimeric receptor (CR) T cells in combination with cetuximab or panitumumab resulted in in vitro anticancer activity in TNBC cells [447]. Cetuximab (IgG1) can induce ADCC- of EGFR-positive cells, whereas panitumumab (IgG2) cannot be due to distinct binding to the different Fc gamma receptors (FcγRs) CD16 and CD32A; IgG2 has low affinity for CD16, while IgG1 and IgG2 bind to CD32A with different affinities [598,599]. The CD32A^131R^-CR T cells were superior to CD16^158F^-CR T cells in their antitumor activity against the EGFR-positive TNBC cell line MDA-MB-468 [447]. The antitumor effects of CD32A^131^-CR T cells in combination with anti-EGFR antibodies were dependent on the level of cell surface EGFR, since this combination does not induce cytotoxicity of MDA-MB-231 cells. The level of EGFR was approximately 2.1-fold higher in MDA-MB-468 cells than in MDA-MB-231 cells [447]. Notably, recently the combination of small-molecule inhibitors and CAR-T cell therapies has been extensively studied in both preclinical and clinical settings [600,601,602,603].

#### 4.5.3. Immune Checkpoint Inhibitors (ICIs)

An immune checkpoint refers to molecules acting as gatekeepers of immune responses [604]. Blocking an immune checkpoint is a promising approach to enhance antitumor immunity. Programmed cell death ligand 1 (PD-L1; also named CD127 or B7-H1) is a ligand for the PD-1 immune checkpoint receptor that inhibits the T-cell effector function in the tumor microenvironment, leading to escape of tumor immunity [605]. PD-L1 expression has been found in 20% of TNBCs and is positively regulated by the PI3K/AKT pathway [606]. The ICI atezolizumab, which is an anti-PD-L1 antibody, in combination with nab–paclitaxel has been approved by the FDA for TNBC treatment [607]. More recently, a pembrolizumab (KEYTRUDA) and chemotherapy (paclitaxel protein-bound, paclitaxel, or gemcitabine plus carboplatin) combination was approved by the US FDA for locally recurrent unresectable or metastatic TNBC [608]. Future studies on the combination of EGFRis and ICIs will be of interest.

### 4.6. Use of EGFR as a Docking Protein for Targeted Drug Delivery

Membrane receptor proteins that are highly expressed in tumors are also potential candidates for therapeutic targeting, imaging, and docking proteins for receptor-targeted delivery systems [609,610]. Drugs can be directly conjugated to the targeting ligand against receptor proteins or encapsulated into receptor-targeted nanocarriers [610]. Due to its high level expression on the surface of various cancers, EGFR has been established as a docking protein for enhanced delivery of therapeutic and diagnostic agents to the tumor stroma with EGFR-overexpressing cancers [248,610,611,612,613,614,615]. For example, approximately 8–80-fold higher expression of EGFR (0.16–1.5 × 10^6^ vs. 1.9 × 10^4^ per cell) has been reported in breast and head and neck cancer cells compared to the normal human milk-derived cells [616,617].

#### 4.6.1. Direct Drug Conjugates

ADCs are composed of monoclonal antibodies directly linked to cytotoxic drugs (payloads) [618,619]. As a fast growing drug classes, currently nine ADCs are approved by the US FDA for cancer treatment [618]. As mentioned earlier, sacituzumab govitecan has been granted accelerated approval for patients with metastatic TNBC [602]. EGFR-targeting ADCs are also being developed (see the recent review [620]). In addition, beyond cytotoxic drugs, various payloads have been conjugated to EGFR-targeting molecules to make ADCs with novel mechanisms, which include photoimmunotheranostics, immunotoxins, cytolytic proteins, and immune modulating drugs (reviewed in [620]). Growing knowledge on the chemistry of the antibodies, linkers, and payloads and their impacts on clinical efficacy can provide further developments of this new modality [618,620].

#### 4.6.2. EGFR-Targeting Nanocarriers

Nanocarriers are nano-sized (10–400 nm) particles that are able to encapsulate therapeutic or diagnostic molecules, which include lipid nanoparticles (liposomes), biocompatible polymers, surfactants, protein particles, RNA nanoparticles, and extracellular vesicles [248,610,611,612,613,614,615,621,622]. A variety of EGFR-targeting molecules have been utilized to effectively and selectively deliver nanocarriers to tumor stroma, including monoclonal antibodies, single-chain antibodies, nanobodies, affimers, affinity peptides, and oligonucleotide aptamers [623,624,625,626,627,628,629,630]. EGFR-targeting nanocarriers can deliver encapsulated drugs more efficiently via receptor-mediated endocytosis; however, escaping lysosomal degradation is a challenging issue that needs to be resolved [631,632,633]. Since nanocarriers are able to encapsulate and deliver insoluble small molecules, antibodies, and nucleic acids such as miRNA and mRNA, EGFR-targeting nanocarriers can provide alternative therapeutic opportunities for patients with TNBC and other EGFR-overexpressing cancers.

## 5. Clinical Studies

A relatively small number of clinical trials have been conducted on the combination of anti-EGFR therapeutics with other drugs. Interestingly, most of the clinical studies of the combinatorial approach have been performed with anti-EGFR antibodies (Table 5), in contrast to preclinical studies (Table 3). Among the 13 studies reported, 10 studies utilized anti-EGFR antibody therapeutics. As mentioned earlier, one disadvantage of anti-EGFR antibodies is that they are slowly infused intravenously over a recommended time period to avoid side effects [38,39]. In contrast, similar to other small-molecule PKIs, EGFR TKIs are orally bioavailable [52]. This advantage of EGFR TKIs provides convenience in designing and dosing in clinical studies and in medicating patients on a daily basis. In addition, multigeneration EGFR TKIs provide wide opportunities considering TNBC genetics or resistance to anticancer drugs.

### 5.1. Anti-EGFR Antibodies in Combination Therapy in Clinical Studies of TNBC

The benefit for patients with TNBC with the combination of cetuximab and paclitaxel was reported in a case report as early as 2007 [634]. A 62-year-old woman was diagnosed with skin metastasis of TNBC after mastectomy followed by adjuvant anthracycline-based chemotherapy and radiotherapy. Various chemotherapies with miltefosine, docetaxel, and vinorelbin + FU failed to treat the metastasized tumor. Surprisingly, a six-course treatment of paclitaxel + cetuximab improved the infiltrating skin metastases [634]. However, the follow-up study failed due to the patient’s return to her country.

Combination treatment with cetuximab and carboplatin for patients with TNBC showed limited outcomes in a phase 2 clinical trial [635]. Compared to the cetuximab monotherapy, cetuximab + carboplatin failed to improve the time to progression (TPP) or OS, with an overall response rate (ORR) < 20% (Table 5)

In addition, unlike the in vitro study [365], a phase 2 clinical study of cetuximab + cisplatin versus cisplatin for patients with TNBC did not meet the primary end point, which was an ORR > 20% [636]. However, cetuximab + cisplatin resulted in a longer PFS and OS (Table 5).

A pilot phase 2 study of a cetuximab and docetaxel combination as a neoadjuvant therapy in stage 2 TNBC showed modest efficacy with manageable toxicity [637]. The pathologic complete response (pCR) rate was 24% and the complete clinical response (cCR) rate was 22% from 25 assessable patients.

In a randomized phase 2 clinical study, an antimicrotubule agent ixabepilone and the combination of ixabepilone and cetuximab resulted in similar clinical activities in the first-line treatment of patients with advanced TNBC [638]. These results did not confirm those of a preclinical study that showed the antitumor efficacy of the gefitinib and ixabepilone combination in the SUM159PT xenografts [406].

Another phase 2 clinical trial involved single-arm treatment with the combination of cetuximab and irinotecan [639]. Among 19 patients, 58% had TNBC. The response rate (RR) of TNBC was 18%, whereas that of non-TNBC was 0%. Although the study was terminated early due to the low RR and rapid disease progression, potentially promising results were noted in patients with TNBC.

Another single-arm study involved the combination of panitumumab with 5-FU + epidoxorubicin + cyclophosphamide (FEC) followed by the combination of panitumumab with docetaxel [640]. The results showed that the treatment was efficacious, with acceptable toxicity. Among 47 assessable patients, the CR was 59.6% (28). A high CD8+ tumor-infiltrating lymphocyte (TIL) count was found to be the strongest predictor for pCR in this study.

A single-arm phase 2 clinical study evaluated a triple combination of the anti-EGFR antibody panitumumab with paclitaxel and carboplatin and showed limited efficacy due to side effects, including neutropenia and thrombocytopenia [641]. Among 14 assessable patients, the ORR was 46% and 2 patients achieved CR after 6 and 9 cycles of therapy. Prolonged neutropenia and thrombocytopenia limited the intended dosing. Similar dose-limiting toxicities have been identified with other EGFRis and platinum-based chemotherapy combinations in patients with NSCLC and metastatic breast cancer [642,643,644].

A single-arm phase 2 study of the combination of panitumumab and neoadjuvant chemotherapy showed the highest pCR rate ever reported in patients with TNBC; however, 10 patients were hospitalized due to treatment-related toxic effects, such as neutropenia, diarrhea, pulmonary embolism, bleeding from the rectum, fever without neutropenia, and confusion of unknown origin [645]. The treatment scheme was relatively complex (Table 5). The expression of p-EGFR and cyclooxygenase-2 (COX2) was correlated with pCR, while no correlations were observed in the expressions of EGFR, E-cadherin, vimentin, and nodal. RNA sequencing analysis revealed the 2 downregulated (*POU3F3* and *EGF1*) and 4 upregulated (*BBOX1*, *GLYATL2*, *MUCL1*, and *LCN2*) genes in samples from patients with TNBC after the first dose of panitumumab treatment. A randomized phase 2 clinical trial (NCT02876107) of the same chemotherapy with and without panitumumab in patients with TNBC is currently under way.

A single-arm phase 2 trial of the combination of panitumumab, gemcitabine, and carboplatin showed no beneficial effect in patients with TNBC [646]. The primary endpoint of median PFS (4.4 months) did not reach the prespecified PFS (5.5 months). No correlation between EGFR amplification, aberrant PI3K pathway activation, or p53 expression and response to this combination has been found.

**Table 5:** Clinical trials of EGFR combination therapy for patients with TNBC.

NCT Number (Publication Year)	EGFRi		Comb Drug		Phase	Clinical Outcomes					Ref
	Treatment				Enrolled						
					Status						
					Sponsor						
NCT00232505(2012)	Cetuximab		Carboplatin		Phase 2			Combo (*n* = 71)		Cetuximab (*n* = 31)	[635]
	QW ^1^, IV ^2^, first: 400 mg/m^2^; subsequently, 250 mg/m^2^		AUC ^3^ of 2 IVs on days 1, 8, and 15 of each 28-day cycle		102						
					Competed	CR ^4^		1 (1%)		0 (0%)	
					Bristol–Myers Squibb	PR ^5^		11 (16%)		2 (6%)	
						SD ^6^		15 (21%)		3 (10%)	
						PD ^7^		38 (54%)		26 (84%)	
						NE ^8^		6 (8%)		0 (0%)	
						TTP 9		2.1 months		1.4 months	
						Median OS ^10^		10.4 months		7.5 months	
NCT00463788 (2013)	Cetuximab		Cisplatin		Phase 2			Combo (*n* = 115)		Cisplatin (*n* = 58)	[636]
	QW, IV, first: 400 mg/m^2^; subsequently, 250 mg/m^2^		Q3W ^11^, IV, 75 mg/m^2^ on day 1, 6 cycles		173						
					Completed	CR		2 (2%)		1 (2%)	
					Merck KgaA	PR		21 (18%)		5 (9%)	
						SD		48 (42%)		18 (31%)	
						PD		34 (30%)		31(53%)	
						NE		10 (9%)		3 (5%))	
						Median PFS ^12^		3.7 months		1.5 months	
						Median OS		12.9 months		9.4 months	
NCT00600249 (2016)	Cetuximab	Docetaxel			Phase 2			Combo (*n* = 25)			[637]
	QW, 18 IVs, first: 400 mg/m^2^; subsequently: 250 mg/m^2^	Q3W, (100 mg/m^2^) on day 1, 6 cycles									
					25						
					Completed	pCR		6 (24%)			
					Merch Serono and Sanofi-Aventis	cCR ^13^		22%			
	
NCT00633464 (2015)	Cetuximab	Ixabepilone			Phase 2			Combo (*n* = 39)		Ixabepilone (*n* = 40)	[638]
	QW, IV, first: 400 mg/m^2^; subsequently, 250 mg/m^2^	Q3W, IV, 400 mg/m^2^			79						
					Completed	CR		0		3 (7.5%)	
					Bristol–Myers Squibb	PR		14 (35.9%)		9 (22.5%)	
						SD		12 (30.8%)		17 (42.5%)	
						PD		10 (25.6%)		9 (22.5%)	
						NE		1 (2.6%)		2 (5.0%)	
						ORR		14 (35.95)		12 (30.0%)	
						Median PFS		4.1 months		4.1 months	
NCT00275041 (2016)	Cetuximab	Irinotecan			Phase 2			Combo (*n* = 19)			[639]
	QW, IV, first: 400 mg/m^2^; subsequently 250 mg/m^2^	IV, 80 mg/m^2^ on days 1 and 8 of a 21-day cycle.									
					19						
					Completed	CR		1			
					NCI and Alliance for Clinical Trials in Oncology	PR		1			
						ORR		11%			
						RR		TNBC, 18% vs. non-TNBC, 0%			
						Median OS		9.4 months			
						Median TTP		1.4 months			
NCT00933517 (2014)	Panitumumab		FEC ^14^ and docetaxel		Phase 2			Combo (*n* = 47)			[640]
					62						
	Q3W, IV, 9 mg/kg, 8 cycles		Q3W, IV, FEC: 500/100/500 mg/m^2^, 4 cycles followed by Q3W, IV docetaxel: 100 mg/m^2^, 4 cycles		Completed	CR		28 (59.6%)			
					Centre Jean Perrin	PR		3 (6.4%)			
						SD		3 (6.4%)			
						Progression		2 (4.3)			
						NE		11 (23.4)			
NCT01009983 (2015)	Panitumumab		Paclitaxel Carboplatin		Phase 2			Combo (*n* = 14)			[641]
	6 mg/kg on days 1 and 15		80 mg/m^2^ paclitaxel and carboplatin AUC of 2 on days 1, 8, 15		14	CR		2 (14.3%)			
					Terminated	PR		4 (28.6%)			
					Wake Forest Univ Health Sci	SD		3 (21.4%)			
						PD		4 (28.6%)			
						NE		1 (7.1%)			
	28-day cycle										
NCT01036087 (2018)	Panitumumab		Nab-paclitaxel + carboplatin		Phase 2			TNBC *n* = 19		HR(+)/HER2(−) *n* = 21	[645]
	1 dose of panitumumab (2.5 mg/kg), then QW, panitumumab (2.5 mg/kg) + nab-paclitaxel (100 mg/m^2^) + carboplatin, 4 cycle followed by Q3W, FU (500 mg/m^2^) + epirubicin (100 mg/m^2^) + cyclophosphamide (500 mg/m^2^), 4 cycles				40	pCR		8 (42.1%)		3 (14.2%)	
					Completed						
					Celgene Corp and Amgen						
NCT00894504 (2016)	Panitumumab		Gemcitabine + Carboplatin		Phase 2			Combo (*n* = 71)			[646]
	Q2W ^15^, IV, 6 mg/kg, 3 cycles		Q2W, IV, Gemcitabine, 1500 mg/m^2^ + Carboplatin, AUC = 2.5 IV, 3 cycles		71	Median PFS		4.4 months			
					Completed	ORR		42%			
					SCRI ^16^, Amgen and Eli Lilly						
NCT00239343 (2011)	Gefitinib		Epirubicin + Cyclophosphamide		Phase 2		Combo (*n* = 71)		Epirubicin + Cyclophosphamide(*n* = 73)		[647]
					144						
	250 mg, daily, 12 weeks		Q3W, epirubicin 90 mg/m^2^ + cyclophosphamide 600 mg/m^2^, 4 cycles		Completed						
					AstraZeneca	pCR	12 (17%)		9 (12%)		
						CR	7 (10%)		7 (10%)		
						PR	41 (58%)		38 (52%)		
						SD	17 (24%)		26 (43%)		
						PD	5 (7%)		2 (2.7%)		
						NE	1 (1.4%)		0 (0%)		
NCT02158507 (2021)	Lapatinib		Veliparib		NA			Combo (*n* = 17)			[648]
	1250 mg, daily, 28 days, starting at cycle 1 day 1			200 mg, every 12 h for 28 days, starting at cycle 1 day 2	23	PR		4 (23.5%)			
					Completed	SD		2 (11.8%)			
					GSK and AbbVie	PD		11 (64.7%)			
	>2 cycles										

^1^ QW, once weekly; ^2^ IV, intravenous; ^3^ AUC, area under the curve; ^4^ CR, complete response; ^5^ PR, partial response; ^6^ SD, stable disease; ^7^ PD, progressive disease; ^8^ NE, non-evaluable; ^9^ TTP, time to progress; ^10^ OS, overall survival; ^11^ Q3W, once every three weeks; ^12^ PFS, progression-free survival; ^13^ cCR, complete clinical response rate; ^14^ FEC, 5-FU + epidoxorubicin + cyclophosphamide; ^15^ Q2W, once every 2 weeks; ^16^ SCRI, SCRI (Sarah Cannon Research Institute) Development Innovations, LLC.

### 5.2. EGFR TKIs in Combination Therapy in Clinical Studies of TNBC

A randomized phase 2 clinical study of gefitinib with neoadjuvant epirubicin and cyclophosphamide (EC) in estrogen-negative breast cancer was not successful. However, post hoc analysis revealed that a significantly higher pCR rate was observed in the TNBC (7/41, 17%) than in the non-TNBC (1/48, 2%) subgroups, but no significant difference was observed between combination therapy (7/41) and EC (5/41) [647].

A phase 1 study of the erlotinib and metformin combination was conducted in 8 patients with TNBC [649]. with a fixed dose of erlotinib of 150 mg daily, a 3 + 3 design of metformin dose escalation was evaluated to determine the dose-limiting toxicities (DLTs). Although no DLTs were observed, no efficacy was demonstrated.

An open-label pilot study of the lapatinib and veliparib combination demonstrated the potential antitumor activity of this combination in patients with advanced TNBC without DLTs [648]. Gene expression analysis revealed an increase in the expression of genes involved in antigen presentation, immune cell infiltration, and cytokine and chemokine signaling. As expected, a reduction was observed in the DNA damage repair genes, such as *BRCA1*, *CRYAB*, and *CKB*. Interestingly, the lapatinib and veliparib combination was reported to have synergistic efficacy in reducing colony formation of TNBC cell lines in vitro [372].

## 6. Conclusions

A wide variety of EGFRi combinations have been successfully applied in TNBC cells preclinically, both in vitro and in vivo (Table 3); however, most of them have not been evaluated clinically yet. To date, no appreciable success of EGFRi combinations has been found in clinical trials (Table 5). Further investigation of the potential of EGFRi combinations should be performed to develop better therapeutics for TNBC in the future.

## Figures and Tables

**Table 3:** Potentiating EGFR inhibition by combination strategies.

Year	EGFRi	Combination Drug		TNBC Cells	Molecules Affected by Combination	Combination Effects		Ref
		Name	Known Target (IC_50_, nM)			Level	Effects	
2004	Gefitinib	Cetuximab	EGFR	MDA-MB-468	-	Cell	- Reduced cell proliferation	[364]
2008	Cetuximab	Cisplatin	DNA	MDA-MB-468	Down: EGFR Up: BRCA1	Cell	- Synergistically induced apoptotic cell death - Depleted EGFR protein	[365]
2009	Gefitinib	Carboplatin + Docetaxel	DNA Microtubule	BT20 HCC9137	-	Cell	- Reduced cell viability - Induced G_2_/M cell cycle arrest	[366]
2011	Erlotinib	Gemcitabine	Antimetabolite	BT-549 MBA-MB-231 MDA-MB-468	-	Cell	- Synergistically reduced cell viability	[11]
2011	Lapatinib	Sirolimus (Rapamycin)	mTOR (~0.1) [367]	MDA-MB-231 MDA-MB-468	Down: p-AKT (S473), p-EGFR (Y1173), p-ERK1/2 (T202/Y204), p-S6 (S235/236)	Cell	- Reduced survival of MDA-MB-231 and MDA-MB-468 but only induced apoptosis of MDA-MB-468 with a concomitant increase of cleaved caspase 3 - Differentially affected p-eIF4E (S209) levels in MDA-MB-468 (down) versus in MDA-MB-231 (up)	[368]
				MDA-MB-231 MDA-MB-468	Down: Ki67 Up: Caspase-3	Xeno ^1^	- Inhibited growth of xenograft tumors of both cells with decreased Ki67 in both tumors and increased apoptosis in MDA-MB-468 tumor	
2011	Cetuximab	IL-2 or IL-15	NK cells	IIB-BR-G IIB-BR-G MT	Up: IFN-γ from NK cells in a co-culture	Cell	- Increased ADCC by NK cells	[369]
2012	Cetuximab	IL-2 or IL-15	NK cells	IIB-BR-G IIB-BR-G MT	Up: CD107a in NK cells in a co-culture	Cell Ex vivo Xeno	- Increased ADCC by NK cells - Reduced tumor volume in xenografts with an increase in the number of infiltrating NK cells in the tumors - No effect on tumor proliferation in xenografts	[370]
2012	Lapatinib	Veliparib (NSC 737664, ABT-888)	PARP2 (2.9), PARP1 (5.2) [371]	MDA-MB-231 MDA-MB-453 MDA-MB-468	Down: nuclear BRCA1, nuclear EGFR Up: cleaved caspase-3, cleaved caspase-9, γ-H2AX	Cell	- Reduced colony formation - Induced apoptosis	[372]
				MDA-MB-231	-	Xeno	- Reduced tumor growth	
2012	Gefitinib	CAT-SKL ^2^	-	MDA-MB-468 SUM149PT	Down: p-EGFR (Y1068)	Cell	- Reduced cell proliferation	[373]
2013	Cetuximab	Dasatinib + Cisplatin	Dasatinib: ABL1 (0.6), SRC (0.8) [374]; KIT^D816V^ (37), KIT (79) [375]	BT20 BT549 MDA-MB-231 SUM102PT SUM149PT SUM229PE	Down: p-AKT (S473), p-EGFR (Y845), p-EGFR (Y1068), p-EGFR (Y1173), p-ERK (T202/Y204) Up: cleaved caspase-9	Cell	- Induced apoptosis - Reduced cell growth and migration	[376]
2013	Gefitinib	PI-103	DNA-PK (2), PI3Kα (8), mTORC1 (20), PI3Kδ (48), mTORC2 (83), PI3Kβ (88), PI3Kγ (150) [377]	MDA-MB-468SUM149PT	Down: p-AKT (S473), BCL2, p-ERK (Y204.Y187), MCL1, XIAP Up: caspase-3/7	Cell	- Reduced cell viability and induced apoptosis in the BL subtype (MDA-MB-468 and SUM149PT) but not in the MSL subtype (HS578T and MDA-MB-231)	[378]
2013	Lapatinib	Bortezomib (Velcade^®^, PS-341, LDP-341, LM341)	20S proteasome (Ki = 0.6) [379]	HS578T MDA-MB-231	Down: BCL2 Up: BAX, Cleaved Caspase-3, Cleaved PARP	Cell	- Reduced colony formation	[380]
				MDA-MB-231	Down: P65 Up: BAX	Xeno	- Reduced tumor growth	
2013	Panitumumab	mAb111	EGFR	BT549	Down: EGFR	Cell	- Reduced in vitro invasion of BT549- Reduced colony formation of HCC70	[381]
				HCC70	-	Xeno	- Reduced tumor volume		
2014	Erlotinib	PHA-665752	MET (9), RON (68), FLK1 (200) [382]	BT20 CRC029 MDA-MB-468	-	Cell	- Reduced cell viability	[150]
2014	Gefitinib	Tepotinib (Tepmetko^®^, EMD1214063)	MET (4) [383]	MDA-MB-468	Down: p-AKT (T308), p-ERK (T202/Y204), p-RPS6 (S235/236)	Cell	- Reduced growth and colony formation	[153]
2014	Duligotuzumab (MEHD7945A)	Ipatasertib (GDC-0068, RG7440)	AKT1 (5), AKT3 (8), AKT2 (18) [384]	HCC70 MDA-MB-468	Down: p-EGFR (Y1068), p-ERK (T202/Y204), p-HER3 (Y1289), p-RPS6 (S240/244)Up: p-AKT (T308)	Cell	- Reduced cell proliferation	[159]
		Pictilisib (GDC-0941, RG7321)	PI3Kα (3), PI3Kδ (3), PI3Kβ (33), PI3Kγ (75), mTOR (Ki = 0.58 μM) [385]		Down: p-AKT (S473), p-AKT (T308), p-EGFR (Y1068), p-ERK (T202/Y204), p-HER3 (Y1289), p-RPS6 (S240/244)	Xeno PDX	- Reduced tumor growth of HCC70 and PDX - Reduced Ki67 index in xenograft tumor	
2014	Gefitinib	Temsirolimus	mTOR (1760) [386]	BT20 MDA-MB-468 MDA-MB-231	Down: p-eIF4B (S422)	Cell	- Reduced cell growth, colony formation, and cell viability - Reduced cap-dependent translation	[387]
2014	Gefitinib	SKI II (SphK-I2)	SPHK (500) [388]	HS578T MDA-MB-231 MDA-MB-436 MDA-MB-468	-	Cell	- Reduced cell proliferation	[389]
						Xeno	- Reduced the growth of MDA-BM-468 xenograft tumor	
2015	Gefitinib	SU11274	MET (10) [390]	HS578T MDA-MB-231	Down: p-AKT (S473), RPS6, p-RPS6 (S235/236)	Cell	- Reduced cell viability and colony formation - No synergistic effect on cell cycle distribution	[15]
2015	Gefitinib	Selumetinib (AZD6244)	MEK1 (14) [391], MEK2 (K*_D_* = 530) [392]	HCC70 MDA-MB-231 MDA-MB-468 SUM149PT SUM159PT	Down: p-AKT (S473), p-ERK (T202/Y204)	Cell	- Induced G_0_/G_1_ cell cycle arrest and apoptosis in SUM149PT	[393]
2015	Gefitinib	Brefeldin A	ARF purified from bovine brain (2 μM) [394], ARF1 (10 μM) [395]	MDA-MB-231	Down: p-AKT, EGFR, p-ERK1/2, HER2, p-SRC	Cell	- Induced cell death	[288]
2015	Lapatinib	Imatinib (Gleevec^®^)	PDGFR (100), KIT (100), ABL1 (600) [396]	HCC1806 MDA-MB-231 SUM159PT	Down: *HOTAIR*, β-catenin, MYC	Cell	- Reduced cell viability	[397]
				MDA-MB-231	-	Xeno	- Reduced tumor growth	
2015	Gefitinib	Ad-wtp53	-	MDA-MB-468	Down: p-AKT (S473) Up: cleaved caspase-3, caspase-9	Cell	- Inhibited cell growth and colony formation - Induced apoptosis and G_2_/M arrest	[398]
						Xeno	- Reduced tumor growth	
2016	Erlotinib	Glesatinib (MGCD265)	DDR2 (1.9), AXL (5.3), MERTK (7.8), PDGFRα (14), VEGFR3 (FLT4) (23), FLT3 (31), MET (46), VEGFR2 (KDR) (66), PDGFRβ (69) [399]	Primary TNBC	Down: p-ERK1/2 (T202/Y204), p-RPS6 (S240/244)	PDX ^3^	- Reduced tumor growth	[400]
		Crizotinib (PF-02341066)	MET (11) [401], ROS1 (Ki < 0.025), ALK (Ki < 0.07), LTK (2.7), FER (3.3), FES (6), PTK2B (FAK2) (14), TNK2 (17), PTK2 (FAK) (17), TRKB (23), TRKA (24), TRKC (46), FRK (PTK5) (53), EGFR^L858R/T790M^ (53), EGFR^T790M^ (56) [402]					
2016	Gefitinib or Erlotinib	Cetuximab or Panitumumab	EGFR	MDA-MB-468 SUM1315MO2	-	Cell	- Induced G_1_ cell cycle arrest	[403]
2016	Gefitinib	GSK269962A	ROCK1 (1.6), ROCK2 (4), MSK1 (49), RSK1 (132) [404]	HCC1806	-	Xeno	- Reduced tumor growth	[405]
				MDA-MB-231	Down: CDK2, Cyclin A, p27, p-RB (S807/811)	Cell	- Inhibited colony formation - Reduced S phase and increased G2 cell cycle arrest	
2016	Cetuximab	Ixabepilone	Microtubule	SUM159PT	Down: LC3B, p62	Cell	- Reduced mammosphere formation efficiency	[406]
					-	Xeno	- Reduced tumor growth	
2017	Cetuximab or panitumumab	Cisplatin or epirubicin		SUM1315MO2	-	Cell	- Induced G_1_ cell cycle arrest compared to a DNA-damaging agent alone that induced G2 cell cycle arrest	[407]
2017	Lapatinib	JNK-IN-8 (JNK Inhibitor XVI)	JNK3 (1), JNK1 (4.7), JNK2 (18.7), KIT^V559D/T670I^ (56), KIT^V559D^ (92) [408]	HCC1569 MDA-MB-231 MDA-MB-436	-	Cell	- Induced apoptosis	[231]
				MDA-MB-231	-	Xeno	- Reduced tumor growth	
2017	Erlotinib or gefitinib	PF573228	FAK1 (4) [409]	BT20 HCC38 HCC1143 HCC1937 MDA-MB-468	Down: p-AKT (S473), p-AKT (T308), HER3, p-S6K (T389), p-STAT3 (Y705)	Cell	- Reduced cell proliferation and colony formation	[410]
		PF431396	FAK1 (2), PYK2 (11) [411]	MDA-MB-468	-	Xeno	- Reduced tumor growth	
2017	Gefitinib	Fingolimod (Gilenya^®^, FTY720)	S1PR (0.033) [412]	HCC1806 MDA-MB-468	Down: CD44, IGFBP3	Cell	- Reduced cell proliferation	[413]
				HCC1806 MDA-MB-468	Down: p-EGFR (Y1068), Ki67 Up: cleaved caspase-3	Xeno	- Inhibited tumor growth and increased mouse survival	
				4T1		Syn ^4^		
2017	Gefitinib	3-methyladenine (NSC 66389)	PIK3C3 (25 μM), PI3Kγ (60 μM) [414]	MDA-MB-231 MDA-MB0465	Down: BCL2 Up: p-ATM (S1981), BAX, cleaved caspase-3, p-CHK1 (S345), p-CHK2 (T68), Cytochrome C, γ-H2AX	Cell	- Reduced cell viability and colony formation - Induced G_0_/G_1_ cell cycle arrest - Induced DNA damage - Promoted mitochondria-dependent apoptosis	[415]
		Bafilomycin A1	H+-ATPase (0.44) [416]	MDA-MB-468	Up: cleaved caspase-3	Xeno	- Reduced tumor growth	
2017	Erlotinib	CAT-SKL	-	HCC70 MDA-MB-468	-	Cell	- Reduced cell viability	[417]
		(−)-epicatechin						
2018	Cetuximab	YM-1	BAG3-HSP70 interaction (4.8 μM) [418]	BT549 MDA-MB-468	-	Cell	- Reduced cell viability	[419]
2018	Gefitinib	MK-2206	AKT1 (5), AKT2 (12), AKT3 (65) [420]	HS578T MDA-MB-231	Down: p-mTOR (S2448), RPS6, p-RPS6 (S235/236), p-RPAS40 (T246), XIAP	Cell	- Reduced cell viability, proliferation, and colony formation - No synergistic effect on cell cycle distribution	[421]
2018	Lapatinib	Foretinib (GSK1363089, XL880)	MET (0.4), VEGFR2/KDR (0.86), TIE2 (1.1), VEGFR3/FLT4 (2.8), RON (3), FLT3 (3.6), PDGFRα (3.6), KIT (3.6), VEGFR1/FLT1 (6.8), PDGFRβ (9.6) [422]	BT549 MDA-MB-231	Down: p-AKT (S473)	Cell	- Reduced cell viability and migration	[423]
2018	Gefitinib	Tepotinib (Tepmetko^®^, EMD1214063)	MET (4) [383]	MDA-MB-468	Down: p-ERK1/2 (T202/Y204), p-STAT3 (Y705)	Cell	- Reduced cell viability and colony formation	[424]
		PF431396	FAK1 (2), PYK2 (11) [411]					
2019	Erlotinib or Gefitinib or Lapatinib	PHA-767491	CDC7 (10), CDK9 (34), GSK3β (220), CDK2 (240), CDK1 (250), CDK5 (460), MAPKAPK2/MK2 (470), PLK1 (980) [425]	BT549 HS578T SKBR7	Down: p-AKT (S473), CDC7, CDK4, Cyclin D1, p-EGFR (T1173), pERK1/2 (T202/Y204), MCM2, p-MCM2 (S40/41), RB, p-RB (S780), POLR2A, p-POLR2A (S2/5),	Cell	- Reduced cell proliferation - Inhibited DNA replication and CDK9-mediated transcriptional elongation - Increased apoptosis and G_2_/M cell cycle arrest	[99]
2019	Osimertinib	Chloroquine (CHQ, Aralen)	-	MDA-MB-231	Up: p-BAD (S112), caspase-3, LC3B-II	Cell	- Reduced cell viability	[426]
2019	Erlotinib	Palbociclib (Ibrance^®^, PD-0332991, LQQ)	CDK4/Cyclin D3 (9), CDK4/Cyclin D1 (11), CDK6/Cyclin D1 (15) [427]	HS578T-MMP17 ^5^ MDA-MB-231-MMP17 MDA-MB-468-MMP17	Down: p-EGFR (Y1068), p-RB (S807/811)	Cell	- Reduced cell proliferation	[428]
				MDA-MB-231-MMP17PDX	Down: Ki67, p-RB (S807/811)	Xeno	- Reduced tumor growth - Reduced MMP17 and p-RB	
2019	AEE788	Everolimus (RAD001)	mTOR (1.6–2.4) [429]	SUM149PT	Down: CDK4, Cyclin B1, Cyclin D1, p-mTOR (S2448)	Cell	- Reduced cell proliferation - Induced apoptosis	[430]
		Sirolimus (Rapamycin)	mTOR (~0.1) [367]					
		Temsirolimus	mTOR (1760) [386]					
2019	Cetuximab	PP2 (AG 1879, AGL 1879)	LCK (4), FYN (5), HCK (5), SRC (100) [431,432]	MDA-MB-231 MDA-MB-468	Down: p-AKT (S473), p-EGFR (Y1173), p-IGF1R (Y1161), p-PI3K, p-SRC (Y416), p-VEGFR2 (Y1175)	Cell	- Reduced cell proliferation	[142]
2020	ABT-414	Navitoclax (ABT-263)	BCL-xL (Ki ≤ 0.5), BCL2 (Ki ≤ 1), BCL2L2 (Ki ≤ 1) [433]	-	-	PDX	- Reduced PDX tumor growth in vivo	[258]
	ABBV-321							
2020	Erlotinib	S63845	MCL1 (Kd = 0.19) [434]	BT20, H38, H1806, H1937, MDA-MB-231, MDA-MB-468,	-	Cell	- Reduced cell viability	[260]
2020	Gefitinib	Crizotinib + Talazoparib	Crizotinib: see above Talazoparib: PARP1 (0.57) [435]	PARPi-resistant SUM149PT	-	Cell	- Reduced cell viability	[436]
2020	Erlotinib	THZ1	CDK7 (3.2) [437]	MDA-MB-231	-	Cell	- Reduced cell proliferation	[438]
2020	Cetuximab	IL-15	NK cells	IIB-BR-G	Up: CD25 and CD69 in NK cells; CD83 and CD86 in DCs; IFN-γ and TNF-α in co-culture supernatant	Cell	- Activated NK cell tumor killing - Stimulated maturation of DCs	[439]
2020	Cetuximab	Dasatinib	ABL1 (0.6), SRC (0.8) [374] KIT^D816V^ (37), KIT (79) [375]	MDA-MB-468	Down: SRC, p-SRC (Y416)	Xeno	- Reduced tumor volume	[440]
2020	Gefitinib	GSK269962A	ROCK1 (1.6), ROCK2 (4), MSK1 (49), RSK1 (132) [404]	MDA-MB-231	Down: p-RPS6 (S235/236) Up: p-AMPK (T172)	Cell	- Increased accumulation of autophagic vacuoles	[339]
				CAL120HCC1806HS578T	Down: p-RPS6 (S235/236) Up: LC3-II			
2020	Afatinib	Dasatinib	ABL1 (0.6), SRC (0.8) [374] KIT^D816V^ (37), KIT (79) [375]	BT20 HCC1937 HDQP1	Down: p-EGFR (Y1068), p-SFKs (Y416), p-SRC (Y527)Up: cleaved caspase-7, p27	Cell	- Reduced cell proliferation- Induced G_1_ cell cycle arrest	[441]
				HCC1806	Down: CDC42, p-EGFR (Y1068)	Xeno	- Reduced tumor growth	
2020	Gefitinib	Everolimus (RAD001)	mTOR (1.6–2.4) [429]	CAL-51	Down: p-4E-BP1 (T37/46), Cyclin B1, Cyclin E1, p-mTOR (S2448)	Cell	- Reduced cell proliferation - Induced G_1_ cell cycle arrest and apoptosis	[442]
2020	Afatinib	Dasatinib + Trametinib (Mekinist^®^, JTP-74057, GSK1120212)	Dasatinib: see above Trametinib: MEK1 (0.92), MEK2 (1.8) [443]	BT20 MDA-MB-468	-	Cell	- Reduced cell proliferation	[444]
2020	Bispecific antibody for EGFR (cetuximab) and HER3 (IgG 3-43)		EGFR (K*_D_* = 21), HER3 (K*_D_* = 19) [445]	MDA-MB-468	Down: p-AKT (T308), p-EGFR (Y1068), p-ERK (T202/Y204), p-HER3 (Y1289) in FaDu, the hypopharyngeal carcinoma cell line	Cell	- Reduced cell proliferation and mammosphere formation	[445]
						Xeno	- Reduced tumor growth with concordant reduction of CSCs	
2020	Pan-HER antibody mixture (combination of 3 sets of 2 antibodies each targeting non-overlapping epitopes of EGFR, HER2, and HER3)		-	15 PDXs	Down: p-AKT (T308), EGFR, p-EGFR (Y1068), p-ERK1/2, p-FAK (Y397), HER3, p-HER3 (Y1289)	PDX	- Reduced tumor growth	[446]
2020	Cetuximab or Panitumumab	CD32A-CR T cells	CD32A^131R^	MDA-MB-468	Up: IFN-γ and TNF-α from CD32A^131R^-CR T cells	Cell	- Induced cancer cell killing	[447]
2020	Afatinib	YM155 (Sepantronium bromide)	*BIRC5*^6^ gene expression (0.54) [448]	-	-	PDX	- Reduced PDX mammary tumor growth *in vivo*	[449]
2020	Cetuximab	Icaritin	-	MDA-MB-231 MDA-MB-436		Cell	- Induced apoptosis- Reduced cell proliferation	[450]
2020	Gefitinib, Erlotinib, or Lapatinib	MK-2206	AKT1 (5), AKT2 (12), AKT3 (65) [420]	MDA-MB-468	Down: p-AKT (S473), p-AKT (T308), p-EGFR (Y1068), p-ERK (T202/Y204), p-PRAS40 (T246), p-RPS6 (S235/236)	Cell	- Reduced cell proliferation (by gefitinib or lapatinib in the presence of MK-2206, AZD8186, or pictilisib)	[451]
		AZD8186	PI3Kβ (4), PI3Kδ (12), PI3Kα (35) [452]			Xeno	- Reduced tumor growth of HCC70 or MDA-MB-468 xenografts (by Erlotinib + AZD8186)	
		Pictilisib (GDC-0941, RG7321)	PI3Kα (3), PI3Kδ (3), PI3Kβ (33), PI3Kγ (75), mTOR (Ki = 0.58 μM) [385]					
2021	Cetuximab	miR-155-5p antagomir	miR-155-5p	MDA-MB-468	Down: BCL2, p-EGFR Up: BAX, cleaved caspase-1, cleaved caspase-3, GSDME, IL-1β	Cell	- Reduced cell proliferation - Increased apoptosis and induced pyroptosis- Reduced migration and invasion	[453]
				MDA-MB-468	Down: p-EGFR, Ki67 Up: cleaved caspase-1, GSDME	Xeno	- Reduced tumor growth - Increased TUNEL-positive cells	
2021	Gefitinib	AT7867 + PD-0325901 (Mirdametinib)	AT7867: AKT2 (17), PKA (20), AKT1 (32), AKT3 (47), p70 S6K (85) [454] PD-0325901: MEK (0.33) [455]	HS578T MDA-MB-231	Down: ERK1/2, GSK3β, p-GSK3β (S9), RPS6, p-RPS6 (S235/236), XIAP Up: cleaved caspase-3	Cell	- Reduced cell proliferation and colony formation- Induced apoptotic cell death	[22]

^1^ Xeno, xenograft; ^2^ CAT-SKL, genetically engineered human catalase with serine-lysine-leucine (SKL) sequence to target peroxisome and 11 arginine peptide transduction domain [373,456]; ^3^ PDX, patient-derived xenograft; ^4^ Syn, syngeneic; ^5^ HS578T-MMP17, MDA-MB-231-MMP17, and MDA-MB-468-MMP17: TNBC cell lines with overexpression of MMP17 (MT4-MMP); ^6^ *BIRC5*, baculoviral IAP repeat-containing protein 5; the gene encoding survivin.

**Table 4:** FDA-approved PKIs that have been experimentally tested with EGFRis in TNBC.

Drug	Primary Target	Initial Year Approval	Company	Approved Indications ^1^	EGFRi Tested
Crizotinib	ALK, ROS1, MET	2011	Pfizer	∙ ALK -positive advanced NSCLC ∙ ROS1-positive NSCLC ∙ ALK-positive anaplastic large cell lymphoma	Erlotinib [400]
Dasatinib	BCR-ABL, SRC	2006	Bristol-Myers Squibb	∙ CML ∙ Philadelphia chromosome (Ph)-positive ALL ∙ Ph-positive CML	Cetuximab [376,440]
Everolimus	mTOR	2009	Novartis	∙ HER2-negative breast cancer, pancreatic neuroendocrine tumors, renal cell carcinoma, angiomyolipoma, subependymal giant cell astrocytoma	AEE788 [430] Gefitinib [442]
Imatinib	BCR-ABL	2002	Novartis	∙ Rare gastrointestinal cancer ∙ ALL	Lapatinib [397]
Palbociclib	CDK4/6	2015	Park Davis	∙ ER- and HER2-positive breast cancer	Erlotinib [428]
Sirolimus	mTOR	1999	Wyeth	∙ Kidney transplant, lymphangioleiomyomatosis	Lapatinib [368]
Selumetinib	MEK1/2	2020	Astra Zeneca	∙ Neurofibromatosis type 1 plexiform neurofibromas	Gefitinib [393]
Temsirolimus	mTOR	2007	Wyeth	∙ Advanced kidney cancer	Gefitinib [387]
Tepotinib	MET	2021	EMD Serono	∙ Metastatic NSCLC with *MET*^ex14^ skipping alterations	Gefitinib [153,424]
Trametinib	MEK1/2	2013	GlaxoSmithKline	∙ Melanoma	Afatinib (with Dasatinib) [444]

^1^https://www.drugs.com/, accessed on 15 April 2021.

## Data Availability

Not applicable.
